# Nur77 improves ovarian function in reproductive aging mice by activating mitophagy and inhibiting apoptosis

**DOI:** 10.1186/s12958-024-01250-6

**Published:** 2024-07-23

**Authors:** Ying Yao, Bin Wang, Kaihua Yu, Ji Song, Liyan Wang, Xuehong Zhang, Yulan Li

**Affiliations:** 1 The First School of Clinical Medicine, Lanzhou University, Lanzhou, China; 2 Reproductive Medicine Center, The First Hospital of Lanzhou University, Lanzhou, China; 3 Key Laboratory for Reproductive Medicine and Embryo of Gansu, No. 1, Donggang West Road, Chengguan District, Lanzhou, Gansu Province China; 4 Department of Anesthesiology, The First Hospital of Lanzhou University, No. 1, Donggang West Road, Chengguan District, Lanzhou, Gansu Province China

**Keywords:** Nur77, Granulosa cells, Aging, PINK1/Parkin, Mitophagy, Apoptosis

## Abstract

Reproductive aging not only affects the fertility and physical and mental health of women but also accelerates the aging process of other organs. There is an urgent need newfor novel mechanisms, targets, and drugs to break the vicious cycle of mitochondrial dysfunction, redox imbalance, and germ cell apoptosis associated with ovarian aging. Autophagy, recognized as a longevity mechanism, has recently become a focal point in anti-aging research. Although mitophagy is a type of autophagy, its role and regulatory mechanisms in ovarian aging, particularly in age-related ovarian function decline, remain unclear. Nerve growth factor inducible gene B (Nur77) is an early response gene that can be stimulated by oxidative stress, DNA damage, metabolism, and inflammation. Recent evidence recommends that decreased expression of Nur77 is associated with age-related myocardial fibrosis, renal dysfunction, and Parkinson's disease; however, its association with ovarian aging has not been studied yet. We herein identified Nur77 as a regulator of germ cell senescence, apoptosis, and mitophagy and found that overexpression of Nur77 can activate mitophagy, improve oxidative stress, reduce apoptosis, and ultimately enhance ovarian reserve in aged mice ovaries. Furthermore, we discovered an association between Nur77 and the AKT pathway through String and molecular docking analyses. Experimental confirmation revealed that the AKT/mTOR signaling pathway is involved in the regulation of Nur77 in ovarian function. In conclusion, our results suggest Nur77 as a promising target for preventing and treating ovarian function decline related to reproductive aging.

**Supplementary Information:**

The online version contains supplementary material available at 10.1186/s12958-024-01250-6.

## Introduction

Reproductive aging is a natural process that typically begins after the age of 30, characterized by a gradual decline in ovarian reserve function, as well as a decrease in both the number and quality of oocytes [1]. When this process manifests before the age of 40, it is referred to as primary or premature ovarian insufficiency [2]. Ovarian aging accelerates the aging of other organs and is accompanied by a series of complications, including fever, night sweats, mood disorders, osteoporosis, cardiovascular disease, and cognitive decline [3]. Worryingly, due to the increasing societal demands on women, more women are choosing to marry and give birth later in life. However, female fertility begins to decline around the age of 32 and rapidly deteriorates after the age of 37, leading to increased oocyte aneuploidy, impaired early embryonic development potential, and a higher spontaneous abortion rate [4]. Despite many women becoming more aware of reproductive health and seeking medical protection, the outcomes are often not ideal.

With the rapid advancement of medical science, the decline in female reproductive capacity is increasingly seen as a condition that can be addressed. The most effective approach is to explore the mechanisms of ovarian aging caused by both internal and external factors. Existing mechanisms, such as oxidative stress, DNA damage, metabolic abnormalities, mitochondrial homeostasis, and autophagy, provide a wealth of research avenues and ideas [5]. Mitochondria are key regulators of energy, metabolism, and redox homeostasis, thereby maintaining cell function. Oocytes are the most mitochondrial-rich somatic cells and depend on these organelles for fertilization and early embryonic development [6]. Mitochondrial damage can release reactive oxygen species (ROS) and cytochrome C and promote inflammasome activation, thus driving germ cell senescence and triggering apoptosis [7].

Autophagy is a crucial health regulator that transports excess or damaged substances from cells to lysosomes for degradation, and it is considered a mechanism for promoting longevity [8, 9]. Mitophagy, a type of autophagy, involves the depolarization of damaged mitochondria, which are recognized by specific receptors on the outer mitochondrial membrane, enveloped into autophagosomes, fused with lysosomes, and subsequently degraded. This process effectively removes damaged or dysfunctional mitochondria, thereby regulating mitochondrial numbers and maintaining normal energy metabolism [10]. However, the role of mitophagy in ovarian function is complex. Spermidine and nicotinamide mononucleotides enhance mitochondrial activity and function in mouse oocytes and granulosa cells by increasing mitophagy, reducing ROS production, and preventing aging-induced apoptosis [11, 12]. Conversely, C-type natriuretic peptide can improve the quality of oocytes in older mothers by inhibiting excessive mitophagy [13]. The association between mitophagy and ovarian function remains unclear due to the limited number of studies, necessitating further research to fully understand and uncover the underlying mechanisms.

Nerve growth factor inducible gene B (Nur77), also known as NR4A1, is a member of the nuclear hormone receptor NR4A family. As an early response gene, Nur77 is involved in the regulation of various physiological and pathological processes, such as cell cycle and apoptosis, lipid metabolism, ischemia and hypoxia stress, and inflammatory stimulation [14]. These factors are related to biological pathways associated with aging. With increasing age, the expression of Nur77 decreases in conditions such as cardiac remodeling and senile kidney disease. In vivo and in vitro experiments have simultaneously shown that Nur77 can target fibrosis signals and decrease cardiac and renal tubulointerstitial fibrosis during aging [15, 16]. Conversely, Nur77 maintains mitochondrial homeostasis by regulating mitophagy and mitochondrial fission in smooth muscle cells, thereby preventing oxidative stress [17]. However, the pathways and molecular mechanisms through which Nur77 participates in ovarian aging remain unclear.

In this study, we initially verified that mitophagy was inhibited in the ovaries of reproductively aging mice, which was accompanied by redox imbalances and elevated apoptosis levels. Notably, our findings indicated that Nur77 expression in aging ovaries declined with age. Additionally, we discovered that overexpression of Nur77 could activate mitophagy via the AKT/mTOR signaling pathway, thus correcting the abnormal number of follicles and sex hormone levels associated with reproductive aging, ultimately improving ovarian function.

## Materials and methods

### Animals

Female C57BL/6 mice (6–8 weeks and 44–48 weeks) were purchased from Lanzhou Veterinary Research Institute and raised under specific pathogen-free conditions with controlled temperature (22 °C ± 1 °C) and humidity (60% ± 10%). They were provided with free access to water and food during a 12-h light/dark cycle. All animal experimental protocols were approved by the Animal Ethics Committee of the First Hospital of Lanzhou University (experimental protocol number: LDYYSZLL2023-13).

### Animals treatment

#### Study I

Young female mice aged 6–8 weeks and naturally aging female mice aged 44–48 weeks were purchased. After one week of acclimatization, blood, and ovarian tissue samples were collected for the relevant tests.

#### Study II

Adeno-associated virus (Genechem Co., Ltd., China) administration for Nur77 overexpression was performed according to the manufacturer's protocol with an EGFP marker. Female mice aged 6–8 weeks were used as the control group, and naturally aging female mice aged 44–48 weeks were randomly divided into three groups: the aging model group, the adeno-associated virus negative control group (AAV-NC), and the adeno-associated virus Nur77 group (AAV-Nur77). The treatment plan was as follows: After 1 week of acclimatization, the natural aging group received no treatment, the AAV-NC group was injected virus in situ with 1.5 mg/kg AAV-NC, and the AAV-Nur77 group was injected virus in situ with the same dose of AAV-Nur77. The day of injection of the virus was considered as day 0 of treatment, followed by continuous observation for 4 weeks. Since the normal estrous cycle of mice is 4–5 days, vaginal smears were performed daily from day 20 of virus transfection for 9 consecutive days until sampling (Fig. 3A). The procedure for in situ virus injection in mice strictly adhered to aseptic operation principles. First, the mice were anesthetized with 1% pentobarbital sodium, and the back was shaved and disinfected with 75% alcohol. A bilateral lower back oblique incision of approximately 0.5 cm was made. The tissue was carefully separated using tissue tweezers, and the ovary was gently pulled out of the abdominal cavity. The virus was slowly injected into the ovary using a 5 μL syringe for about 3 min, causing the ovarian capsule to swell and whiten. After the injection, the needle puncture site was compressed with sterile gauze for 2–3 min for hemostasis. The ovary was gently returned to its original position, and the wound was sutured. After 4 weeks of AAV-Nur77 treatment, all animals were euthanized under anesthesia, and samples were collected for further experiments.

### Vaginal smears and estrous cycle determination

Vaginal smears were obtained using the saline douching method daily from 08:00 to 09:00. A drop of mouse vaginal lavage was placed onto a slide, spread evenly to approximately 1.5 × 2 cm, and allowed to air dry naturally. Following hematoxylin and eosin (H&E) staining, a microscopic examination (Olympus, Japan) was conducted to observe cell types and morphology, determining the regularity of the mice's estrous cycle. Normal estrous cycle phenomena are described as follows: Proestrus, characterized by predominantly irregularly shaped nucleated epithelial cells with few leukocytes and keratinized epithelial cells; Estrus, characterized by almost all lamellar anucleated keratinized cells; Metestrus, characterized by a small number of non-nucleated keratinized epithelial cells with leukocytes comprising the majority of cells; and Diestrus, characterized by crinkled epithelial cells and a large number of leukocytes in the smear.

### H&E staining and masson's trichrome

Ovary tissues were excised, fixed in a 4% paraformaldehyde fixing solution, and embedded in paraffin. Sections of 5 μm thickness were obtained and subjected to H&E staining and Masson staining following standard protocols. After H&E staining, the follicles were classified and counted to assess the effect of Nur77. Briefly, ovaries were serially sectioned at 5 μm intervals, and every 10th section was stained with H&E. Follicle classification was performed according to the Pederson standard. The number of follicles at each stage was estimated by counting every 10th section and multiplying the sum by a correction factor of 10 to represent the entire ovary [18].

### Enzyme-linked immunosorbent assay (ELISA)

After the completion of administration, the blood samples were collected from the mice's orbital sinus under anesthesia with pentobarbital sodium. After clotting at room temperature for 90 min, serum was obtained by centrifugation at 1800 × g and stored at -80 °C for further analysis. The serum levels of estradiol (E_2_), anti-Müllerian hormone (AMH), and follicle-stimulating hormone (FSH) were analyzed using the standard protocols of ELISA kits (FANKEW, China).

### Malondialdehyde (MDA) and superoxide dismutase (SOD) assays

Ovarian tissue lysates were prepared with lysis buffer for the Western and IP Kit (Boster, China) or with PBS, all kept on ice. Homogenates of ovarian tissues obtained by brief oscillation were centrifuged at 2000 × g for 10 min at 4 °C, and the supernatants were collected for further experiments. The concentrations of supernatants were determined using the Bicinchoninic Acid (BCA) Protein Assay Kit (Thermo, USA). The MDA levels were measured using the Lipid Peroxidation MDA Assay Kit (Solarbio, China) and calculated by a standard curve according to the kit's manufacturer's instructions. The SOD activity was detected using the Total SOD Assay Kit with WST-8 (Beyotime, China) and calculated according to the manufacturer's instructions. All experiments were independently repeated three times.

### Western blot (WB) analysis

Protein extraction was performed on ovarian tissues, which were subsequently lysed and isolated. Protein levels were then measured using a BCA Protein Assay Kit (Boster, China). Equal amounts of denatured protein were separated by electrophoresis in 10% SDS polyacrylamide gels and then transferred to polyvinyl difluoride membranes (Millipore, Billerica, USA). Next, these membranes were saturated with a blocking buffer for 1 h. Following this, membranes were incubated with rabbit polyclonal anti-Nur77 (1:1,000 dilution; Abcam), rabbit polyclonal anti-p16 (1:1,000 dilution; Abcam), rabbit polyclonal anti-p21 antibodies (1:2,000 dilution; Proteintech), rabbit polyclonal anti-Parkin antibodies (1:1,000 dilution; Abcam), and mouse monoclonal anti-PINK1 antibodies (1:500 dilution; Abcam) at 4 °C. Then, the blots were incubated with HRP-conjugated anti-rabbit IgG for 2 h. Finally, proteins were detected using the enhanced chemiluminescence (ECL) detection kit (Bio-Rad, USA) and visualized using film exposure. The gels and western blots were analyzed with ImageJ.

### Immunofluorescence (IF)

Paraffin-embedded ovary sections on the same side were dewaxed and dehydrated in different concentrations of ethanol and then incubated with 3% H2O2. Subsequently, they were blocked with 5% BSA for 1–2 h at room temperature and incubated with the primary antibody against Nur77 (1:100 dilution; Abcam), P16 (1:100 dilution; Proteintech), H2AX (1:100 dilution; MCE), or LC3 (1:200 dilution; Proteintech) at 4 °C. The slices were then incubated with the secondary antibody at room temperature for 60 min, washed, and treated with an antifade mounting medium containing DAPI (Solarbio, China).

### Terminal-deoxynucleotidyl transferase-mediated nick end labeling (TUNEL)

A TUNEL cell apoptosis detection kit (Servicebio, China) was used to assess apoptosis in ovarian sections. The analysis was performed according to the manufacturer’s instructions. Briefly, after being permeabilized, the sample was incubated with the TUNEL detection solution. The nucleus was labeled with DAPI. Finally, the samples were analyzed via fluorescence microscopy (Olympus, Japan).

#### Real-time quantitative polymerase chain reaction (PCR)

Total RNA was extracted from ovarian tissues with TRIzol reagent (Invitrogen Life Technologies, USA) and reverse transcribed to create cDNA using the Takara reagent kit (Takara Biotechnology, Japan) following standard methodology. SYBR Green master mix (Tiangen, China) was used for quantitative PCR amplification. Relative gene expression was normalized against GAPDH. The primers used were as follows:

**Table:** 

	Forward	Reverse
Nur77	AGCTTGGGTGTTGATGTTCC	TAAAGGCACATGGGTGACAG
PINK1	GGGCTTGCCAATCCCTTCT	CAGGCGATCATCTTGTCCAAT
Parkin	TCTTCCAGTGTAACCACCGTC	GGCAGGGAGTAGCCAAGTT
p62	GACAAGAGTAACACTCAGCCAAGCA	CTCCATCTGTTCCTCTGGCTGTC
LC3II	CCGTCCGAGAAGACCTTCAA	TCTTGCGGCAGGAGAACCTA
p53	TGCTCCGATGGTGATGGC	TTCCAGATACTCGGGATACAAATTT
p21	GGGACAAGAGGCCCAGTACT	CAATCTGCGCTTGGAGTGA
p16	GGCTCTGCTCTTGGGATTGG	ATCGTGCGATATTTGCGTTCC
H2AX	TTGATTGCCGGGCTTAGAGG	CTGCGGCAGGTATAGAACTC
Bax	GCCTTTTTGCTACAGGGTTTCAT	TATTGCTGTCCAGTTCATCTCCA
Bcl2	TGACTTCTCTCGTCGCTACCGT	CCTGAAGAGTTCCTCCACCACC
β-actin	GTGACGTTGACATCCGTAAAGA	GTAACAGTCCGCCTAGAAGCAC

#### Transmission *electron* microscopy (TEM)

Ovaries were fixed in TEM fixative (Servicebio, China) and dehydrated in gradient alcohol and acetone at room temperature. After polymerization with resin, the ovaries were cut to a thickness of 80 nm on an ultramicrotome. Finally, the samples were observed by TEM (HT7700, Japan).

#### Network construction and molecular docking

The protein–protein interaction (PPI) network of Nur77 was constructed using the STRING database and visualized with Cytoscape 3.8. Additionally, the protein models used for docking were Nur77 (PDB ID: 2QW4) and AKT (PDB ID: 1H10). The HDOCK SERVER (http://hdock.phys.hust.edu.cn/) was employed for molecular docking. Nur77 was selected as the receptor protein, and AKT was chosen as the ligand–protein. Pre-treatment of the proteins, including deletion of water molecules and excess ligands, and addition of hydrogen atoms, was completed using PyMol 2.4. The model with the lowest binding energy was selected as the best docking model, and PyMOL was used to visualize PPIs.

## Results

### Decreased expression of Nur77 in reproductive aging mice's ovaries

**Fig. 1:**
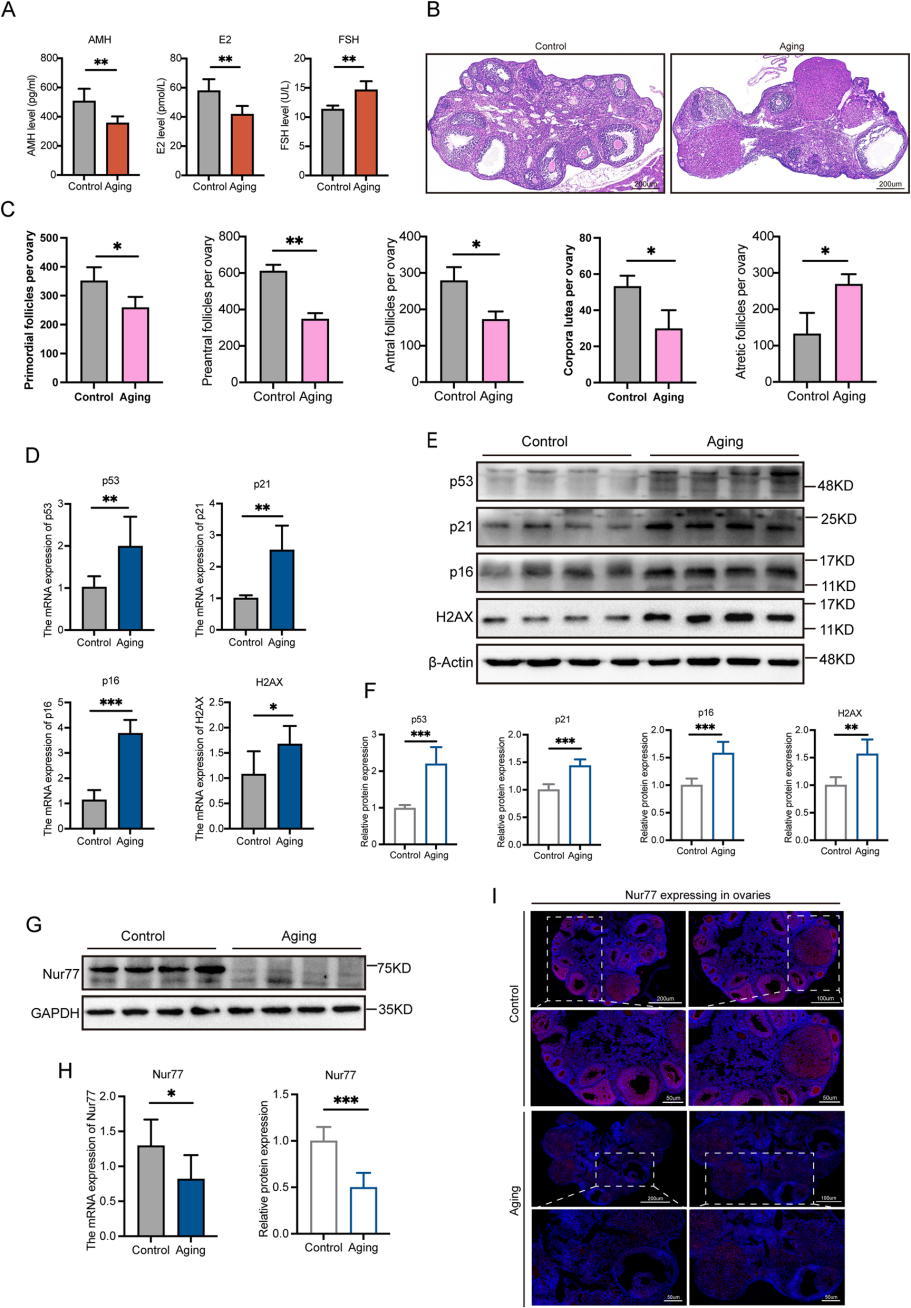
Ovarian function and Nur77 expression decreased in aged mice. **A** Comparison of serum sex hormones AMH, E_2_, and FSH between the control group and aging group. **B** and **C** Representative images of H&E staining of ovaries tissues, and follicle count in control group and aging group mice. **D** The mRNA expression levels of p53, p21, p16 and H2AX between the control group and aging group. **E** and **F** The protein expression levels of p53, p21, p16 and H2AX between the control group and aging group. **G** and **H** The mRNA and protein expression levels of Nur77 between the control group and aging group. **I** Immunofluorescence staining was used to detect the localization and expression of Nur77 in the ovarian tissues of the control group and aging group. Scale: 200 μm, 100 μm, 50 μm. Error bars, mean ± SEM. *n* = 6–8 per group. *P < 0.05, **P < 0.01, ***P < 0.001

To accurately evaluate Nur77 expression in reproductive aging ovaries, we first assessed ovarian function in the control and experimental groups (aging group). We observed E_2_ and AMH decreased, while FSH increased (Fig. 1A). Additionally, a decrease in the number of ovarian primordial follicles, antral follicles, and corpus luteum in the aging group, along with an increase in atresia follicles (Fig. 1B and C), indicating decreased ovarian function.We also detected increased expression levels of the aging indicators p53, p21, p16, and the DNA oxidative damage indicator H2AX (Fig. 1D-F) by PCR and WB in the experimental group. Meantime, we observed a significant decrease in Nur77 gene and protein expression levels in the ovaries of aged mice (Fig. 1G and H). IF also showed decreased expression of Nur77 (Fig. 1I).

### Decreased activity of mitophagy and increased level of apoptosis in the ovaries of reproductively aging mice

**Fig. 2:**
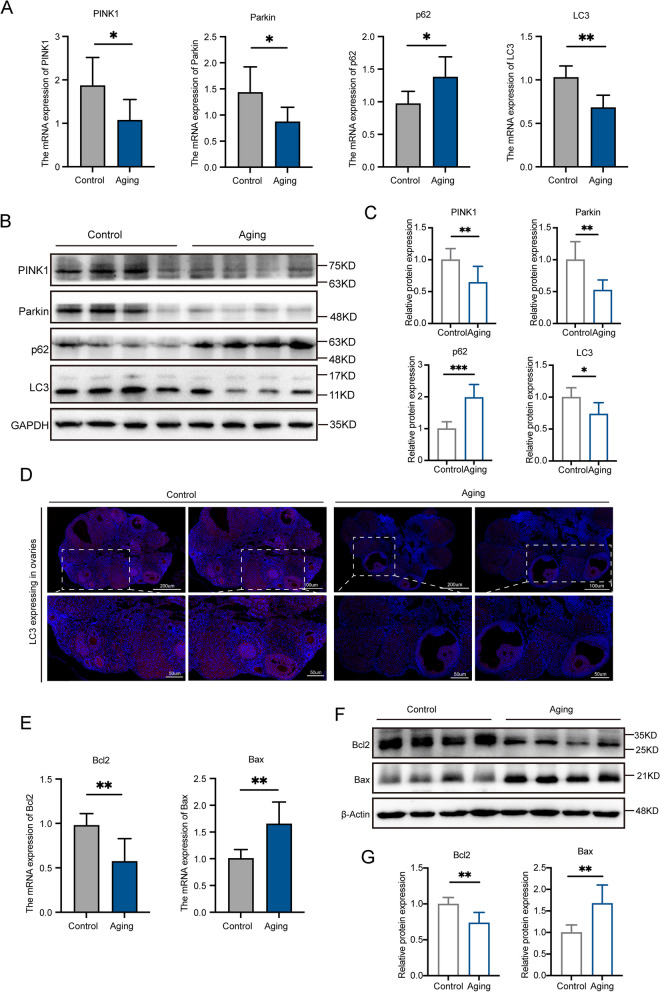
The activity of mitophagy decreased and the level of apoptosis increased in the ovary of reproductively aging mice. **A** The mRNA expression levels of LC3, p62, PINK1 and Parkin between the control group and aging group. **B** and **C** The protein expression levels of LC3, p62, PINK1 and Parkin between the control group and aging group. **D** Immunofluorescence staining was used to detect the localization and expression of LC3 in the ovarian tissues of the control group and aging group. Scale: 200 μm, 100 μm, 50 μm. **E** The mRNA expression levels of Bcl2 and Bax between the control group and aging group. **B** and **C** The protein expression levels of Bcl2 and Bax between the control group and aging group. Error bars, mean ± SEM. *n* = 6–8 per group. *P < 0.05, **P < 0.01

The expression level of mitophagy in the ovary of reproductively aging mice is uncertain. Therefore, we observed mitophagy activity in two sets of comparative experiments. We found that the expression level of the mitophagy classic index genes (PINK1/Parkin/LC3) in the ovaries of old mice decreased, while p62 expression increased (Fig. 2A). The protein expression level showed similar changes (Fig. 2B and C), indicating decreased mitophagy activity in ovarian tissues. LC3, a marker for autophagy, helps autophagosomes and lysosomes join together to break down damaged mitochondria [19]. Therefore, we used IF to detect LC3 expression and found a significant reduction in LC3 expression in the ovaries of reproductive aging mice (Fig. 2D). Additionally, increased Bax expression and decreased Bcl2 expression at the gene and protein levels in the experimental group's ovaries indicate an elevated apoptosis rate of germ cells in aging ovaries (Fig. 2E-G), which is consistent with decreased ovarian function due to abnormal hormone secretion caused by germ cell apoptosis [20].

### Nur77 increased the reproductive aging mice's ovarian index and improved their estrous cycle

**Fig. 3:**
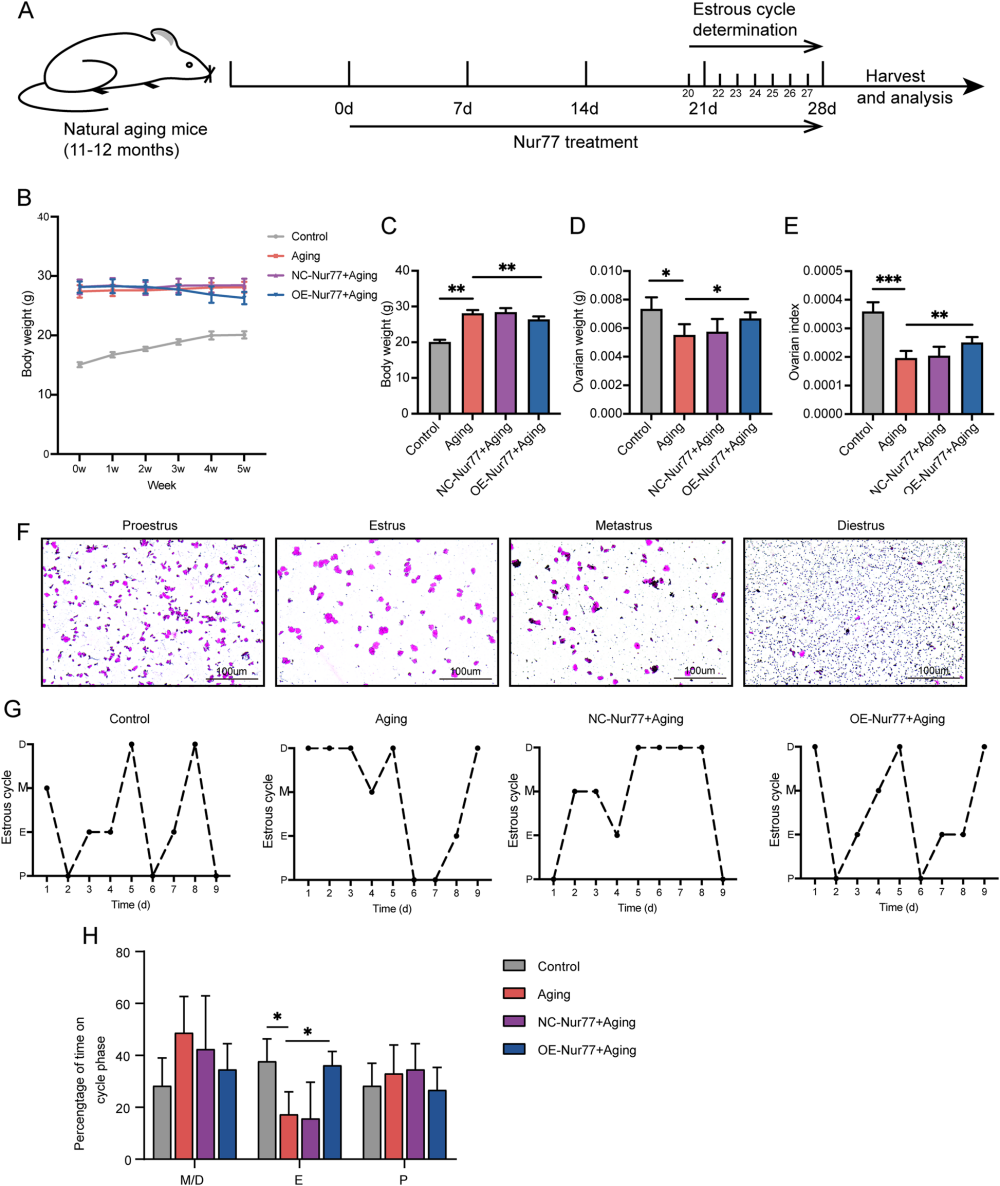
Nur77 increased the reproductive aging mice's ovarian index and improved their estrous cycle. **A** Treatment timeline of Nur77 in aging mice. **B** The changes in body weight of mice in each group after overexpression of Nur77. **C** The comparison of the body weights of mice in each group at the end of the study. **D** and **E** Ovarian weight and ovarian index of mice in each group after overexpression of Nur77. **F** Characteristics of vaginal smears in different stages of the estrous cycle in mice. Scale: 100 μm. **G** Representative estrous cycles. ( proestrus, estrus, metestrus and diestrus. **H** Quantitative analysis of time on the cycle phase in 9 days. Error bars, mean ± SEM. *n* = 6–8 per group. *P < 0.05, **P < 0.01, ***P < 0.001

To investigate whether Nur77 can improve ovarian function in reproductive aging mice, we used a natural aging mouse model to simulate the progressive decline of ovarian function with physiological age. Figure 3A demonstrates the treatment timeline for Nur77. Aged mice typically exhibit obesity, ovarian atrophy, and decreased ovarian function [21]. Throughout the experiment, we monitored the changes in body weight of mice in each group. We observed that the body weight of mice in the control group gradually increased, consistent with the growth trend. The body weight of mice in the natural aging group and the virus-negative control group did not change significantly, but the body weight of mice in the OE-Nur77 + aging group decreased at the end of the study compared with their initial body weight (Fig. 3B and C). The ovarian weight and index of aging mice in the Nur77 overexpression group significantly increased (Fig. 3D and E). Additionally, to evaluate the stages of the estrous cycle, we performed vaginal examinations and vaginal smears [22]. Figure 3F shows representative smears of the four stages of the mouse estrous cycle (proestrus, estrus, metestrus, and diestrus). Normally, the mouse estrous cycle lasts 4–5 days, with any single cycle lasting more than 3 days considered abnormal [22]. We continuously observed the vaginal smears of mice from each group for 9 days and found that the estrous cycle of mice in the control group was normal (Fig. 3G), whereas that of mice in the aging group and NC-AAV + aging group was abnormal and irregular. However, overexpression of Nur77 significantly improved the estrous cycle disorder in aged mice (Fig. 3G). Comparing the total proportion of the four stages of the estrous cycle in each group, we found that overexpression of Nur77 significantly increased the estrus (E) than metestrus and diestrus (M/D) or proestrus (P).

### Nur77 improves follicular development and sex hormone levels in the ovaries of reproductive aging mice

**Fig. 4:**
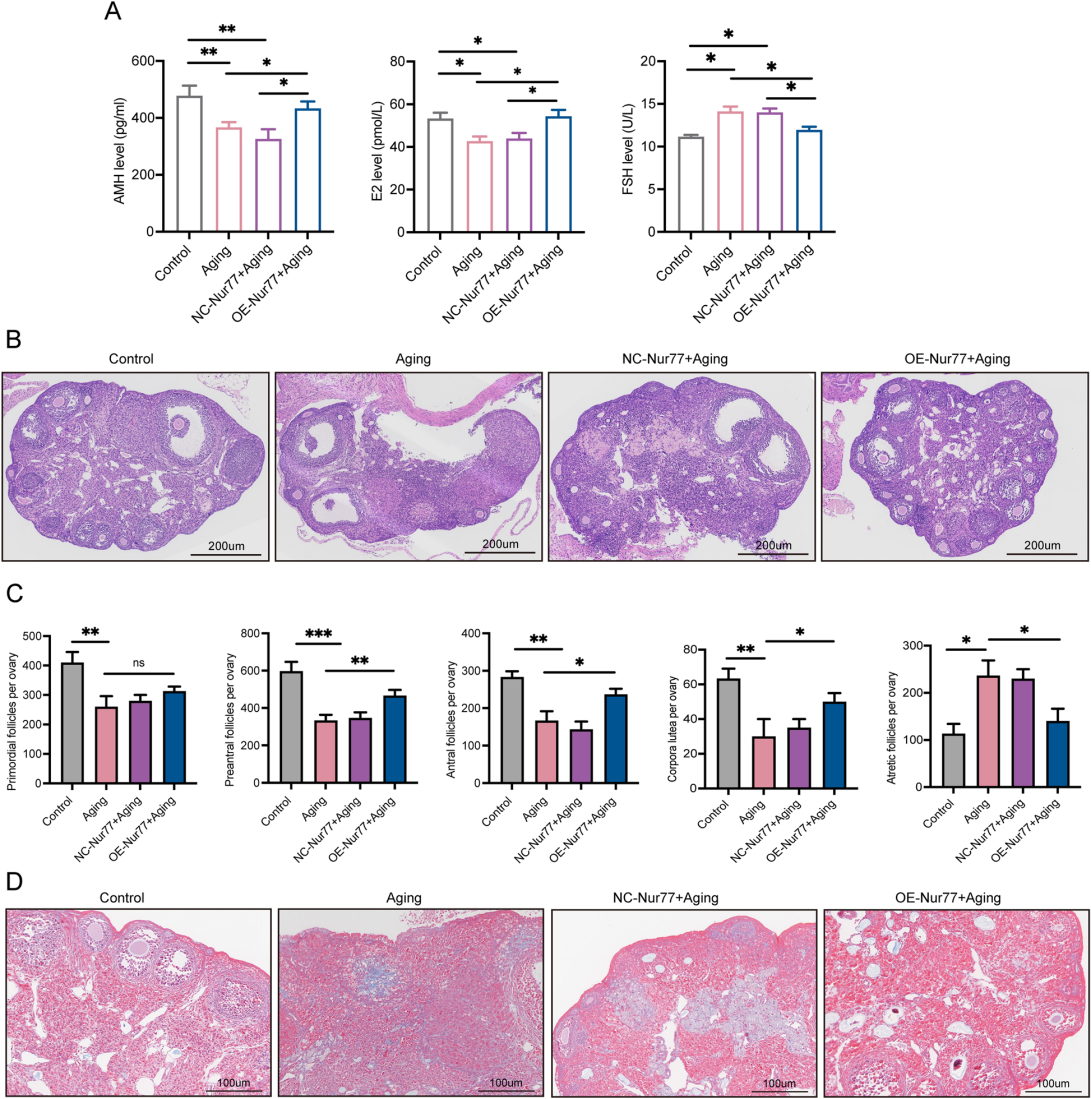
Nur77 improves follicular development and sex hormone levels in the ovaries of reproductive aging mice. **A** Comparison of serum sex hormones AMH, E_2_, and FSH in each group after overexpression of Nur77. **B** and **C** Representative images of H&E staining of ovaries tissues, and follicle count in each group after overexpression of Nur77. Scale: 200 μm. **D** Representative images of masson staining of ovaries tissues in each group after overexpression of Nur77. Scale: 200 μm. Error bars, mean ± SEM. *n* = 6–8 per group. *P < 0.05, **P < 0.01, ***P < 0.001

Clinical evaluation of ovarian function typically involves the use of serum sex hormone levels and basic antral follicle count as indicators [23]. In this experiment, the levels of FSH, AMH, and E_2_ in serum were measured by ELISA (Fig. 4A). The level of FSH was higher and the levels of AMH and E_2_ were significantly lower in the serum of aging mice than in those of the controls (*P* < 0.001), indicating poor ovarian function. However, the OE-Nur77 + aging group showed a significant improvement in sex hormone levels. Ovarian granulosa cells, which develop to a certain extent and act as important supporting cells for follicular development, secrete sex hormones. The improvement in sex hormone levels suggests an increase in follicular development. Excitingly, H&E staining (Fig. 4B and C) revealed that overexpression of Nur77 significantly increased the number of preantral and antral follicles and decreased the number of atretic follicles in aged mice. Additionally, the increase in the number of corpus luteum indicates that Nur77 can promote follicular ovulation. Furthermore, ovarian fibrosis increases with age, hindering follicular development and ovulation [24]. Aged mice exhibited more ovarian fibrosis than the control group, as indicated by Masson staining (Fig. 4D), while high expression of Nur77 reduced ovarian fibrosis, possibly contributing to ovulation and improve ovarian function.

### Nur77 reduces oxidative stress and apoptosis in the ovaries of reproductively aging mice

**Fig. 5:**
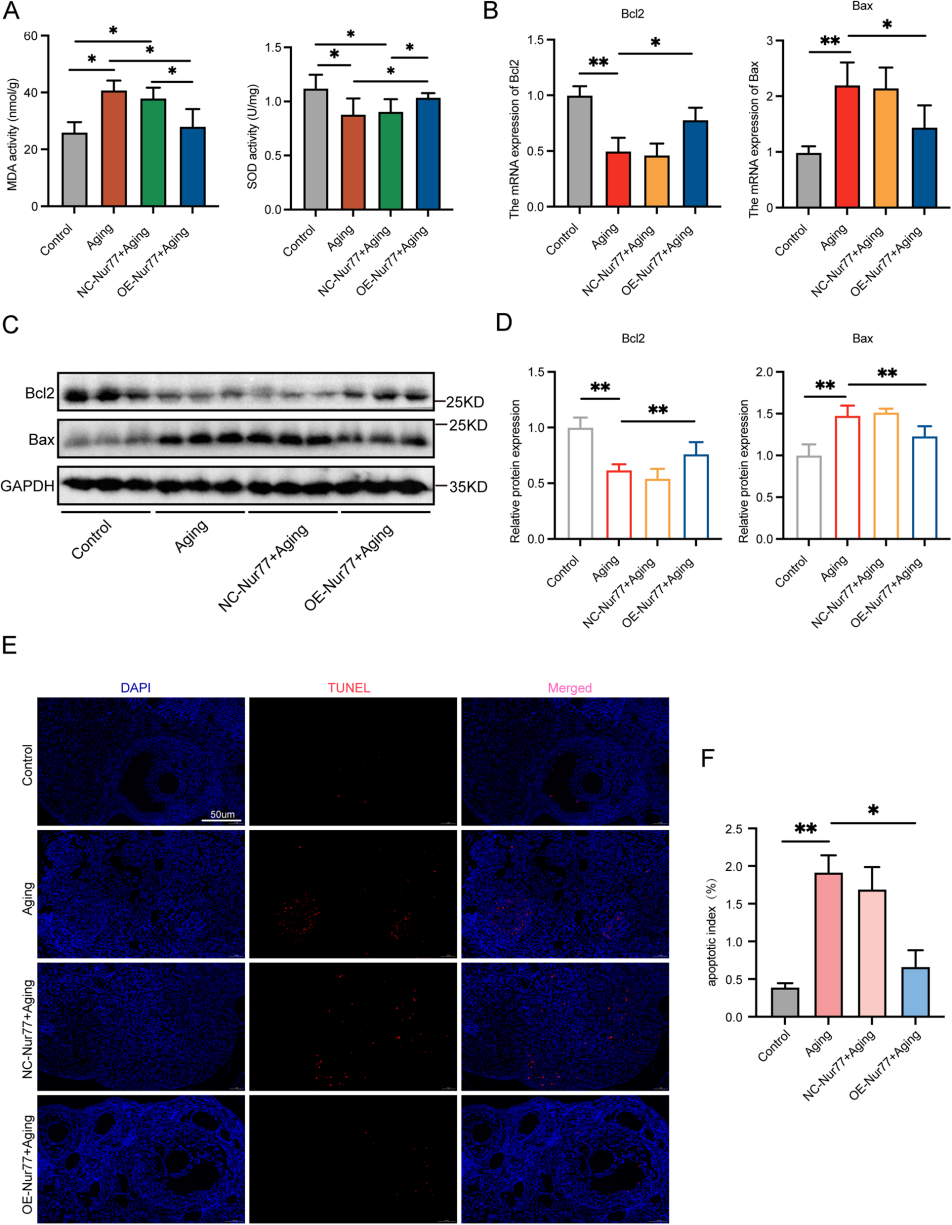
Nur77 reduced oxidative stress and apoptosis in the ovaries of reproductively aging mice. **A** The levels of SOD and MDA in ovarian tissue of each group after Nur77 overexpression. **B** The mRNA expression levels of Bcl2 and Bax in each group after overexpression of Nur77. **C** and **D** The protein expression levels of Bcl2 and Bax in each group after overexpression of Nur77. **E** and **F** Representative images of TUNEL staining of ovaries tissues, and statistical analysis in each group after overexpression of Nur77. Scale: 50 μm. Error bars, mean ± SEM. *n* = 6–8 per group. *P < 0.05, **P < 0.01

The metabolic capacity of senescent cells decreases with age, leading to persistent redox imbalance in senescent tissues [25]. To assess the oxidative stress level in ovarian tissue, we measured the levels of SOD and MDA (Fig. 5A). MDA increased and SOD decreased in the ovaries of aged mice. However, overexpression of Nur77 in aged mice significantly improved redox disorders. Oxidative stress and mitochondrial dysfunction often form a vicious cycle, promoting each other and potentially leading to apoptosis. Germ cell apoptosis can significantly impact follicle growth and development. Our study found that overexpression of Nur77 significantly reduced the expression of the pro-apoptotic gene Bax and increased the expression of the anti-apoptotic gene Bcl2 in the ovaries of aged mice (Fig. 5B), with protein detection results consistent with this (Fig. 5C and D). Additionally, to visually assess apoptosis, we used TUNEL to evaluate the effect of Nur77 on ovarian cell apoptosis (Fig. 5E-F). The aging group showed a significant increase in red fluorescence in ovarian granulosa cells and ovarian interstitial cells (indicating TUNEL positivity), indicating an increase in the apoptosis rate. However, the OE-Nur77 + aging group exhibited little red fluorescence, indicating a significant decrease in the apoptosis rate.

### Nur77 slows down ovarian aging in reproductive aging mice

**Fig. 6:**
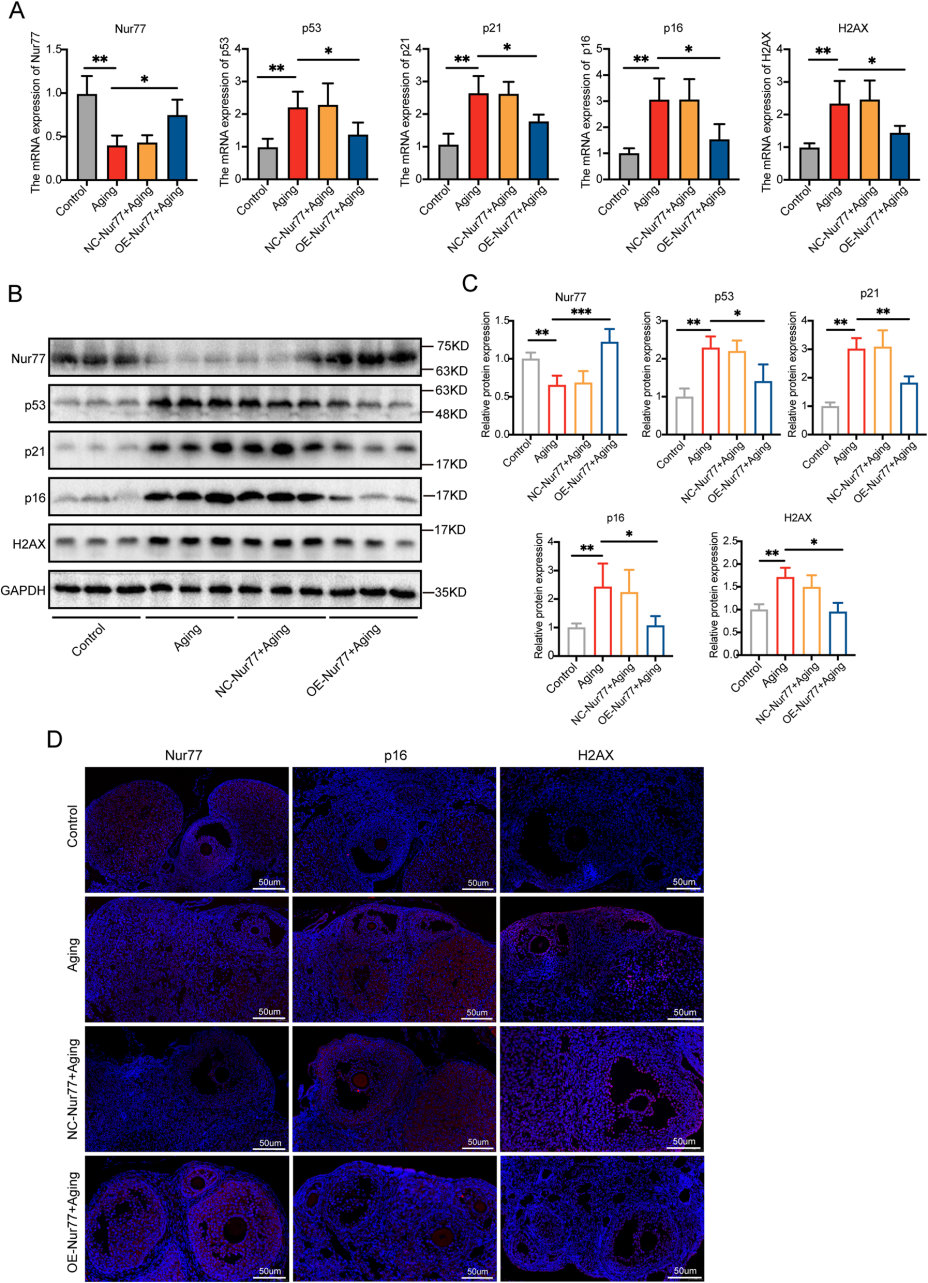
Nur77 slows down ovarian aging in reproductive aging mice. **A** The mRNA expression levels of Nur77, p53, p21, p16 and H2AX in each group after overexpression of Nur77. **B** and **C** The protein expression levels of Nur77, p53, p21, p16 and H2AX in each group after overexpression of Nur77. **D** Immunofluorescence staining was used to detect the localization and expression of Nur77, p16, H2AX in the ovarian tissues of the each group after overexpression of Nur77. Scale: 50 μm. Error bars, mean ± SEM. *n* = 6–8 per group. *P < 0.05, **P < 0.01, ***P < 0.001

Oxidative stress and cell senescence are mutually reinforcing factors. As Nur77 expression decreases with age, p53 acetylation increases, leading to increased levels of the cell cycle arrest protein p21 and exacerbation of oxidative stress and DNA damage responses [26]. Our findings indicate that Nur77 can mitigate the redox imbalance in aged mice and reduce the apoptosis rate of germ cells. To further assess the impact of Nur77 on ovarian aging in mice, we injected adeno-associated virus into the mice's ovaries in situ. PCR and WB analysis confirmed significantly higher Nur77 expression levels in the ovaries of aged mice injected with lentivirus (Fig. 6A–C). Subsequent examination of DNA damage response indicators (H2AX) and cell senescence indicators (p53, p21, and p16) in the ovaries of each group of mice revealed significantly higher expression levels in aged mice compared with the control group. However, overexpression of Nur77 significantly reduced the expression levels of these aging indicators (Fig. 6A–C). Immunofluorescence analysis further demonstrated increased expression of Nur77 and decreased expression of H2AX and p16 in the ovaries of aged mice, confirming the role of Nur77 in ameliorating ovarian aging (Fig. 6D).

### Nur77 activates mitophagy in the ovaries of reproductive aging mice

**Fig. 7:**
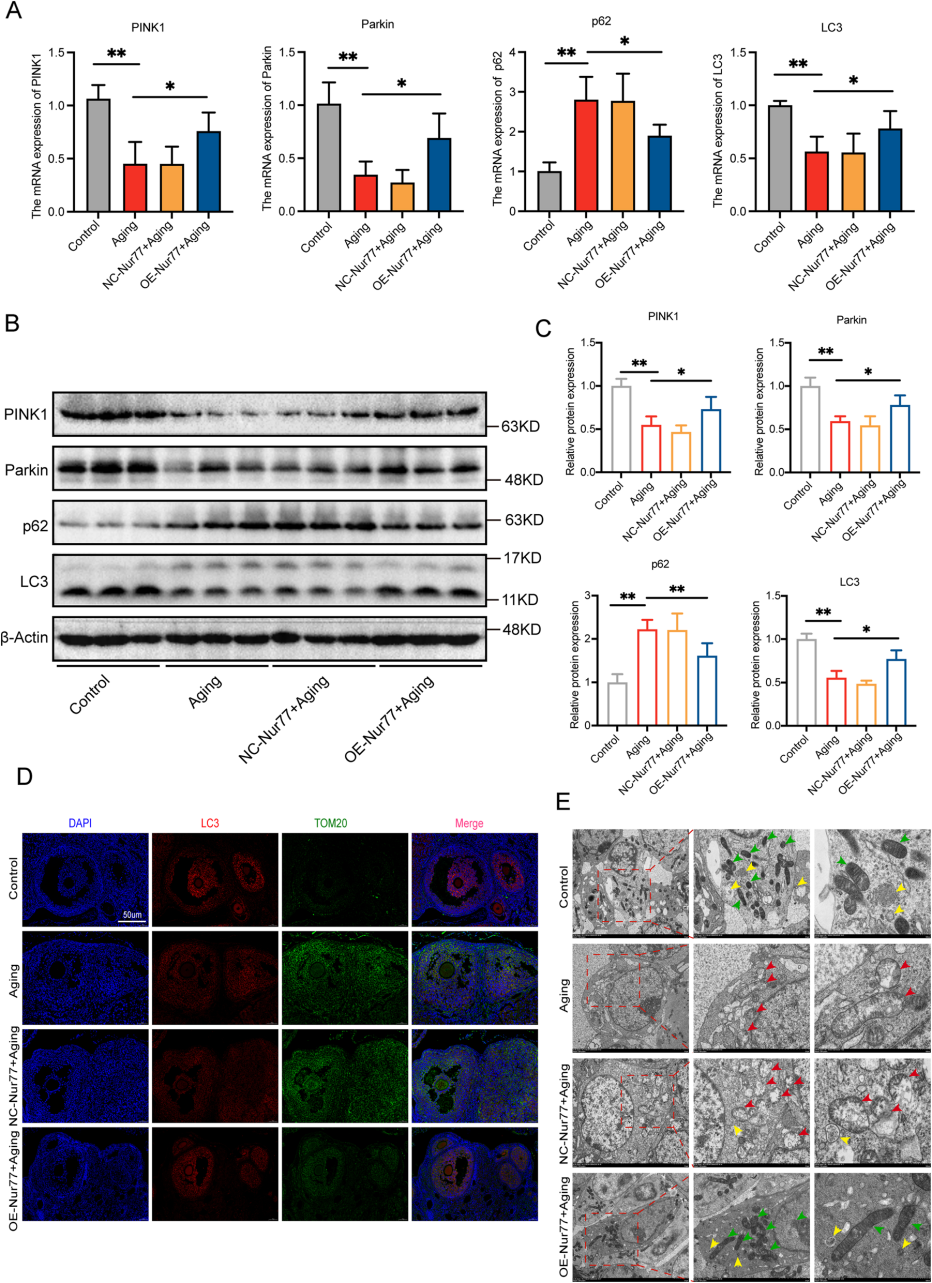
Nur77 activates mitophagy in the ovaries of reproductive aging mice. **A** The mRNA expression levels of LC3, p62, Parkin and PINK1 in each group after overexpression of Nur77. **B** and **C** The protein expression levels of LC3, p62, Parkin and PINK1 in each group after overexpression of Nur77. **D** Immunofluorescence staining was used to detect the localization and expression of LC3 AND TOM20 in the ovarian tissues of the each group after overexpression of Nur77. Scale: 50 μm. **E** Electron micrographs showed the normal mitochondrial morphology (green arrow), mitochondrial damage (red arrow), and mitophagy body formation (yellow arrow), in ovarian tissues. Scale: 2.0 μm, 500 nm. Error bars, mean ± SEM. *n* = 6–8 per group. *P < 0.05, **P < 0.01, ***P < 0.001

The initial stage of our experiments revealed a decrease in mitophagy activity in the ovaries of aged mice. Previous studies have suggested that reduced mitophagy activity can lead to ovarian dysfunction [11]. Given that Nur77 can alleviate ovarian aging and improve ovarian function, we hypothesized that Nur77 might achieve this by activating mitophagy. To test this hypothesis, we first used PCR (Fig. 7A) and WB (Fig. 7B and C) to assess the expression levels of mitophagy indicators in the ovaries of each group. In the OE-Nur77 + aging group, the expression of PINK1, Parkin, and LC3 increased, while the expression of p62 decreased compared with the aging group and the NC-Nur77 + aging group. Additionally, translocase of outer mitochondrial membrane 20 (TOMM20), an important component of mitochondrial outer membrane proteins, was evaluated using LC3 and TOMM20 double IF to assess mitophagy levels (Fig. 7D). Compared with the control group, the aging group showed decreased LC3 and increased TOM20, indicating reduced mitophagy activity. However, overexpression of Nur77 reversed this effect, suggesting that Nur77 activates inhibited mitophagy in the aging ovary. The functional association between mitophagy and mitochondrial dynamics is crucial for mitochondrial homeostasis [27]. The number of normal mitochondria is critical for germ cell development. Under TEM (Fig. 7E), ovarian granulosa cells in the aging group exhibited damaged mitochondrial structure (red arrow), increased vacuoles in the mitochondrial matrix, fragmented and swollen mitochondria, lost cristae, and no apparent mitochondrial autophagosomes. In contrast, the OE-Nur77 + aging group showed a significant increase in the number of normal mitochondria (green arrow), along with the formation of autophagosomes and mitochondrial autophagosomes (yellow arrow). This provides a more tangible explanation for Nur77's positive regulation of mitophagy.

### Nur77 improves ovarian aging in reproductively aging mice through the AKT/mTOR pathway

**Fig. 8:**
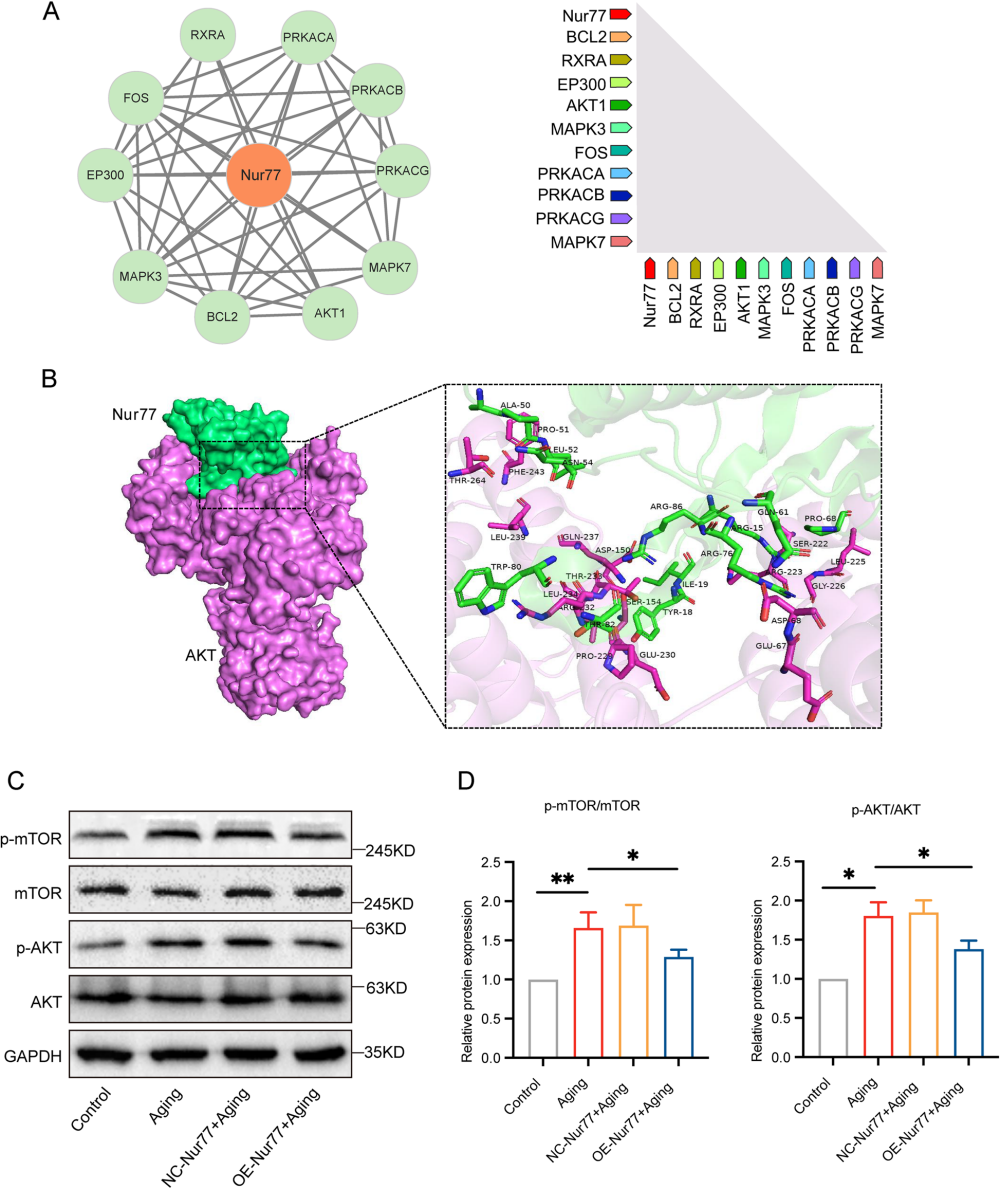
Nur77 improves ovarian aging in reproductively aging mice through AKT/mTOR pathway. **A** PPI network was constructed by String, hub genes were identified, and predicted functional partners. **B** Molecular docking analysis. Nur77 was selected as the receptor protein, and AKT was selected as the ligand protein. Blue represents the hydrogen bond. Nur77 is green, and AKT is purple. **C** and **D** The protein expression levels of p-mTOR/mTOR and p-AKT/AKT in each group after overexpression of Nur77. Error bars, mean ± SEM. *n* = 6–8 per group. *P < 0.05, **P < 0.01

We constructed a PPI network and identified hub genes using STRING analysis. The main hub genes were Bcl2, RXRA, and EP300, with AKT and MAPK closely associated with the Nur77 pathway (Fig. 8A). Consequently, we selected the AKT pathway for further verification. Utilizing HDOCK SERVER, we performed protein–protein molecular docking of Nur77 and AKT, yielding a docking score of − 283.76, confidence score of 0.9355, and ligand RMSD of 83.93, indicating stable binding between the two proteins (Fig. 8B). Subsequently, we used WB to assess the protein expression levels of AKT/p-AKT and mTOR/p-mTOR (Fig. 8C and D). Compared with the control group, the expression of phosphorylated AKT and mTOR in ovarian tissue of the aging group significantly increased. However, this effect was reversed after Nur77 overexpression, indicating that Nur77 exerts its effects by activating the AKT/mTOR pathway. Studies have shown that inhibition of the AKT/mTOR pathway can activate autophagy and mitophagy, thereby alleviating UV-induced skin photoaging and hepatocyte senescence in alcoholic fatty liver mice [28, 29]. This finding supports our hypothesis that Nur77 can delay reproductive aging in mice by activating mitophagy, possibly through the AKT/mTOR pathway.

## Discussion

Reproductive aging is a continuous natural phenomenon characterized by a limited number of follicles that can mature and ovulate in a woman's ovary [30]. Additionally, changes in women's social roles have led many to miss the optimal reproductive age, and endocrine abnormalities and chronic complications caused by ovarian dysfunction result in significant mental and psychological distress. Currently, common drugs like metformin, visfatin, sphingosine 1-phosphate, and dehydroepiandrosterone, along with treatments like stem cell therapy, autologous mitochondrial transfer, and hyperbaric oxygen therapy, have shown some progress in delaying ovarian aging. However, due to the complexity of treatment implementation and the unclear mechanisms underlying ovarian aging, these approaches still face many deficiencies and controversies [31]. Therefore, further research is essential to explore the mechanisms and targets involved, aiming to improve relevant treatments.

Nur77 can function as a ligand-independent transcription factor that regulates various biological processes, including inflammation, oxidative stress, and metabolic abnormalities [32]. Researchers have found that a decrease in Nur77 in age-dependent diseases can accelerate the development of cardiac and renal fibrosis, as well as Parkinson's disease [15, 16, 33]. Based on this, we propose to further explore whether Nur77 plays a role in age-dependent ovarian aging. Mice are the primary model organisms for ovarian aging experiments [34]. Mice aged 10–12 months correspond to women aged 36–38 years [35, 36]. At this age, mice exhibit a decline in fertility, but it has not yet been completely lost. To simulate the critical age period when women seek fertility and experience conception difficulties, and to ensure clinical relevance, we selected mice around 11 months of age as the research subjects.

Ovarian reserve, a key term in assessing ovarian function, is evaluated clinically using indicators such as antral follicle count and serum sex hormone levels including AMH, FSH, and E_2_. As women age, the number of antral follicles decreases, leading to reduced E_2_ secretion and increased FSH levels. AMH, primarily secreted by antral follicles, remains relatively stable throughout the menstrual cycle, making it a valuable predictor of ovarian function [37, 38]. Various mouse models, including natural aging and induced pathological aging, demonstrate declines in ovarian function characterized by decreased E_2_ and AMH, and increased FSH levels [39, 40]. Our study observed similar changes in aged mice, and Nur77 overexpression led to decreased FSH and increased E_2_ and AMH levels. Follicular development is closely linked to sex hormone levels, and Nur77 has been implicated in androgen signaling and angiogenesis during follicle development, essential for normal ovarian function [41, 42].

Our findings indicate that Nur77 overexpression not only increased the ovarian index and reduced the number of atretic follicles in aged mice but also promoted the development and ovulation of preantral and antral follicles. The number of age-related follicles determines the onset of menstrual cycle disorders and ultimately, menopause [43]. The estrous cycle in mice resembles the menstrual cycle in females. After sexual maturity, ovarian hormones drive cyclical changes in the vaginal mucosa of mice, reflecting the ovary's functional status [44]. Normally, the estrous cycle in female mice lasts 4–5 days. Cycles shorter than 4 days, longer than 6 days, or a single period lasting more than 3 consecutive days are considered estrous cycle disorders. The decline in ovarian function in aging mice leads to estrous cycle disorders, a phenomenon we also observed. However, we noted a partial restoration of the sexual cycle in mice overexpressing Nur77. These findings suggest that Nur77 can regulate ovarian endocrine function and maintain ovarian reserve.

In the normal reproductive cycle of women, a delicate balance between ROS and antioxidants is maintained. Excessive ROS and an imbalance in this system lead to oxidative stress, a primary cause of ovarian aging. Oxidative stress not only accelerates ovarian aging but also exacerbates other aging processes, including telomere shortening, mitochondrial dysfunction, apoptosis, and inflammation [25].SOD and MDA are key players in cellular oxidative stress and free radical reactions [45]. Our study observed an increase in MDA and a decrease in SOD in the ovaries of aged mice, indicating redox abnormalities. Prolonged oxidative stress can lead to DNA damage, reflected by increased H2AX expression. The tumor suppressor protein p53 plays a central role in DNA damage response, while p16 and p21 are important regulators of cellular senescence [46].

Nur77 overexpression led to an increase in the expression of SOD and a decrease in the expression of H2AX, p53, p21, and p16 in the ovaries of aged mice, indicating a reduction in oxidative stress and cellular senescence. Previous studies have suggested that the expression of p16 is inversely proportional to the number of follicles, promoting cell senescence and apoptosis [47]. Additionally, p53 can induce the expression of apoptosis-related genes like Bax and Bcl2 [48]. Germ cell apoptosis is directly related to ovarian function, and our results showed that Nur77 overexpression reduced the expression of pro-apoptotic gene Bax and increased the expression of anti-apoptotic gene Bcl2, leading to a decrease in the apoptosis rate. Overall, these findings suggest that Nur77 alleviates oxidative stress and delays age-dependent ovarian function decline in aged mice. By targeting oxidative stress and apoptosis, Nur77 may offer a promising approach to preserving ovarian function and fertility in women.

Mitochondria play a crucial role in supporting the high-energy demands of germ cells and are involved in various cellular processes such as metabolic precursor synthesis, steroid hormone synthesis, calcium regulation, ROS production, and apoptosis [48, 49]. Therefore, maintaining mitochondrial quality control, including mitochondrial biogenesis and mitophagy, is essential for the health of germ cells. Mitochondrial autophagy, or mitophagy, is a specialized form of autophagy responsible for degrading and removing damaged mitochondria [50]. The process typically involves the PINK1/Parkin signaling pathway, a well-known pathway for mitophagy initiation. When mitochondria are damaged, PINK1 accumulates on their depolarized outer membrane, recruiting and activating Parkin to ubiquitinate mitochondrial proteins. This ubiquitin signal marks the damaged mitochondria for recognition by autophagy cargo receptors, such as p62/SQSTM1, which then deliver them to autophagosomes for degradation. The interaction between p62 and LC3, a protein involved in autophagosome formation, facilitates the clearance of damaged mitochondria [51].

In our study, we observed that the levels of PINK1, Parkin, and LC3 in the ovaries of aged mice decreased, while p62 levels increased. Additionally, TEM revealed swollen and vacuolated mitochondria with shortened or sparse cristae and reduced presence of mitochondrial autophagosomes in the ovarian granulosa cells of elderly mice. In contrast, the control group showed both autophagosomes and normal mitochondrial structure. These findings suggest a decline in mitophagy activity associated with ovarian aging. Previous studies have demonstrated that enhancing mitophagy in oocytes and granulosa cells during reproductive aging can improve ovarian function [11, 12]. Based on these findings, we hypothesized and confirmed that Nur77 may alleviate ovarian dysfunction by regulating mitophagy.

Through various experimental methods, including gene and protein analysis, double IF, and TEM, we demonstrated that overexpressing Nur77 in the ovaries of aged mice can activate PINK1/Parkin-mediated mitophagy. Additionally, we investigated the involvement of classical signaling pathways such as PI3K/AKT/mTOR and MAPK/ERK in the autophagy of ovarian granulosa cells and oocytes, which can influence the formation and quality of primordial follicles and germ cells [52]. We used String to construct a PPI network and identified a close relationship between Nur77 and the activation of AKT and MAPK pathways. Using the HDOCK SERVER, we conducted protein–protein molecular docking and confirmed a stable combination between Nur77 and AKT. Subsequent experiments were conducted to verify these findings, indicating that the AKT/mTOR signaling pathway may play a role in Nur77's regulation of mitophagy in reproductive aging ovaries.

Although our experiment has made significant progress, some limitations need to be addressed. First, we focused solely on the classic PINK1/Parkin signaling pathway for mitophagy regulation. However, this is not the only mechanism involved in mitophagy, and future research should explore additional pathways to provide a more comprehensive understanding of mitophagy regulation in ovarian aging. Additionally, we did not include a recovery experiment using mitochondrial autophagy inhibitors to inhibit mitochondrial autophagy in conjunction with Nur77 overexpression at the beginning of the experiment.

## Conclusion

**Fig. 9:**
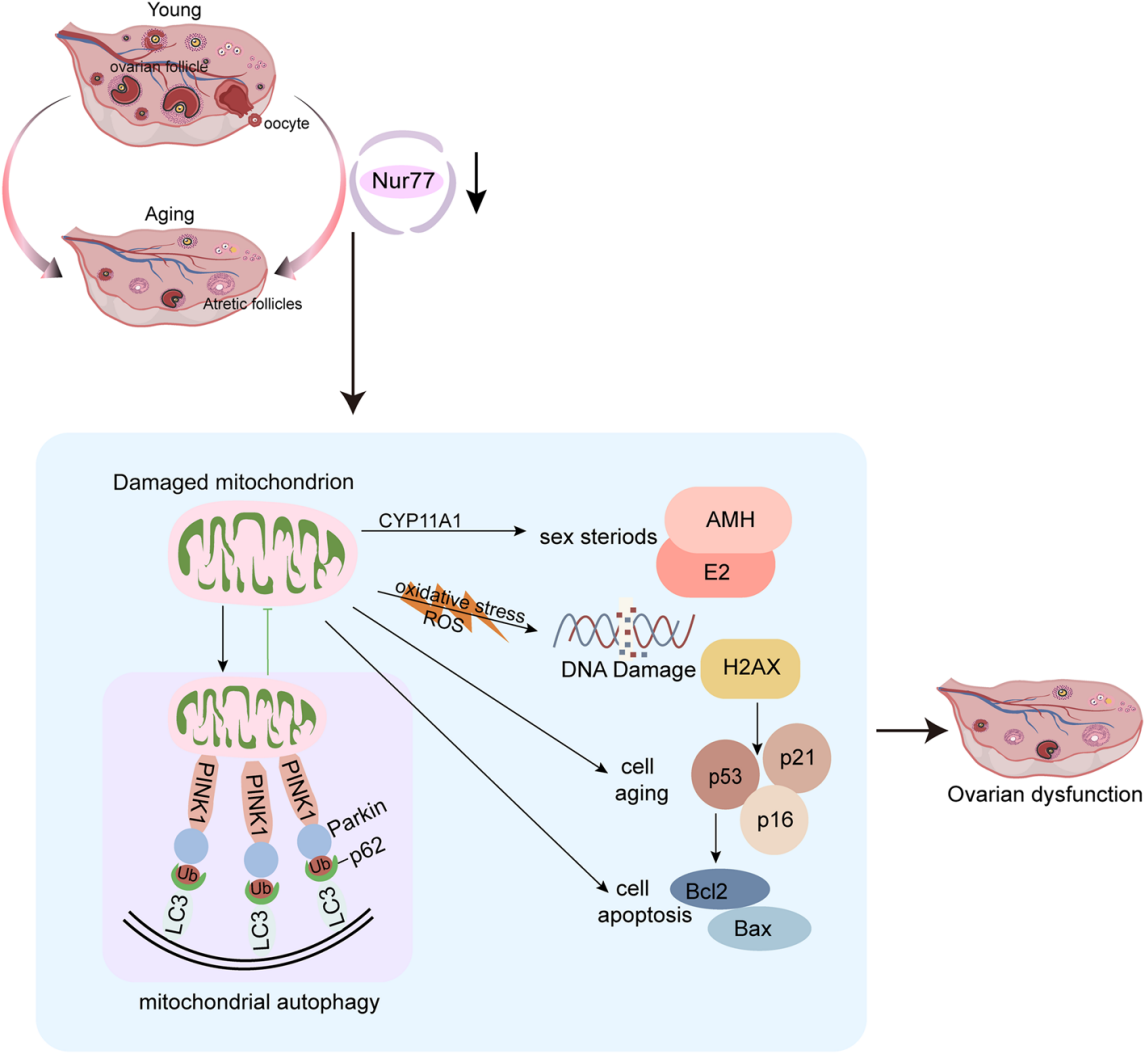
The schematic diagram of the regulation of Nur77 in ovarian aging

In summary, despite these limitations, our study demonstrates that Nur77 effectively inhibits oxidative stress, apoptosis, and ovarian damage in reproductive aging ovaries, ultimately improving ovarian function by promoting mitophagy (Fig. 9). These findings suggest Nur77 is a promising target for the prevention and treatment of reproductive aging and related diseases.

### Supplementary Information

Supplementary Material 1.

## Data Availability

The underlying data that supports the findings of this study will be made available upon reasonable request to the corresponding author. This is done in the spirit of open science and allows others in the field to verify our results and use the data for further explorations.
