# Supplementary figures and images for: Nur77 improves ovarian function in reproductive aging mice by activating mitophagy and inhibiting apoptosis
Source: Reprod Biol Endocrinol. 2024 Jul 23;22:86. doi: 10.1186/s12958-024-01250-6 (PMC11265396; doi:10.1186/s12958-024-01250-6)

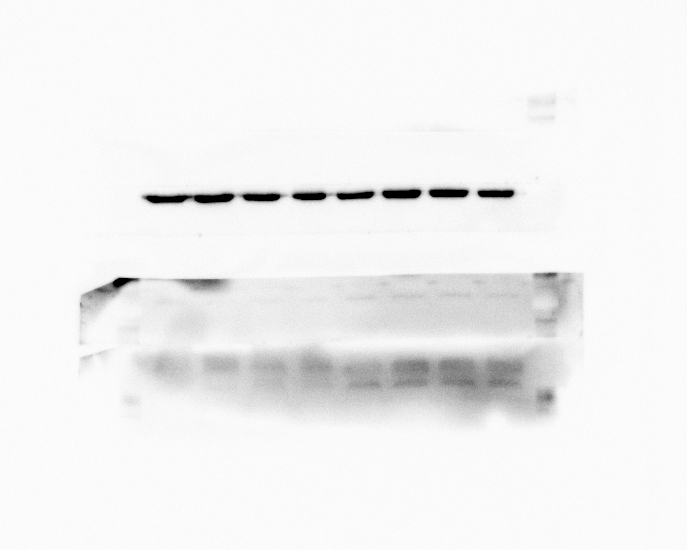

Supplement: Supplementary file 1 — Supplementary Material 1. [file 12958_2024_1250_MOESM1_ESM.zip › WB original picture/fig1-Nur77-p53-p21-p16-H2AX/Actin/Actin sample pattern.tif]

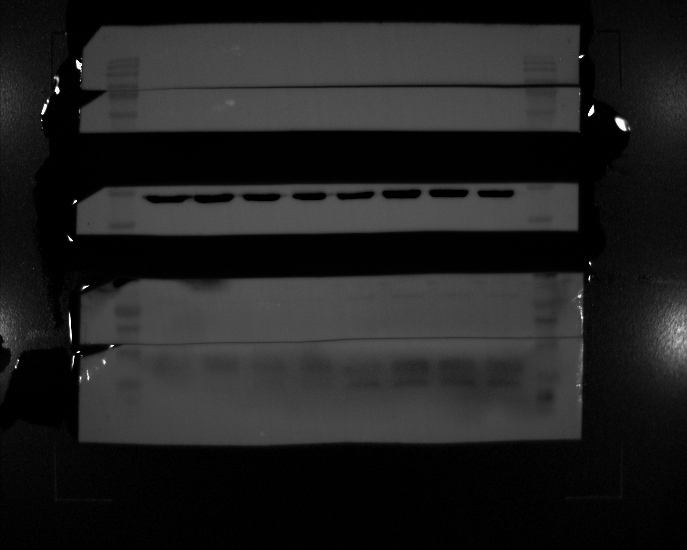

Supplement: Supplementary file 1 — Supplementary Material 1. [file 12958_2024_1250_MOESM1_ESM.zip › WB original picture/fig1-Nur77-p53-p21-p16-H2AX/Actin/Actin superposed graph.tif]

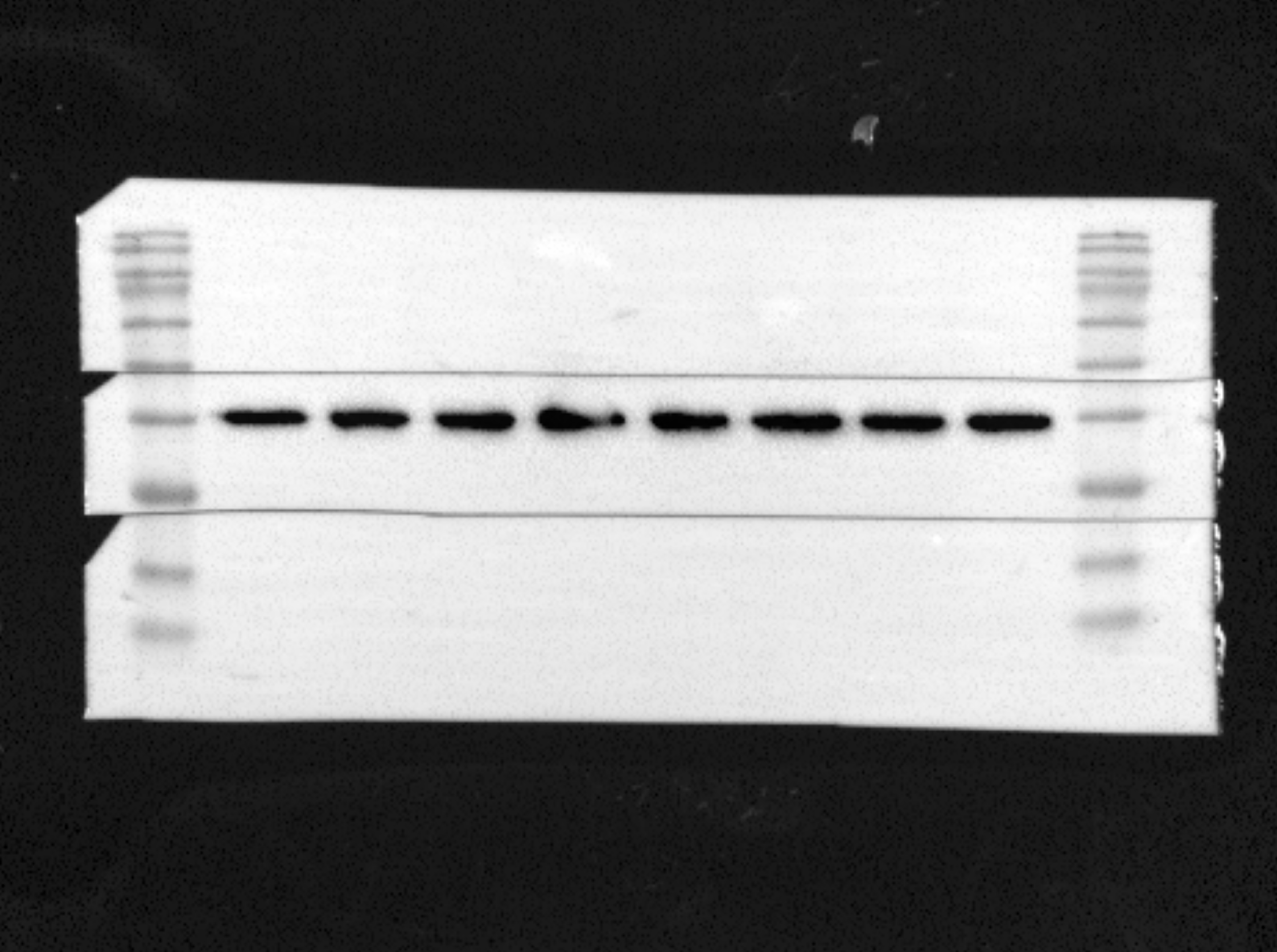

Supplement: Supplementary file 1 — Supplementary Material 1. [file 12958_2024_1250_MOESM1_ESM.zip › WB original picture/fig1-Nur77-p53-p21-p16-H2AX/GAPDH/GAPDH.tif]

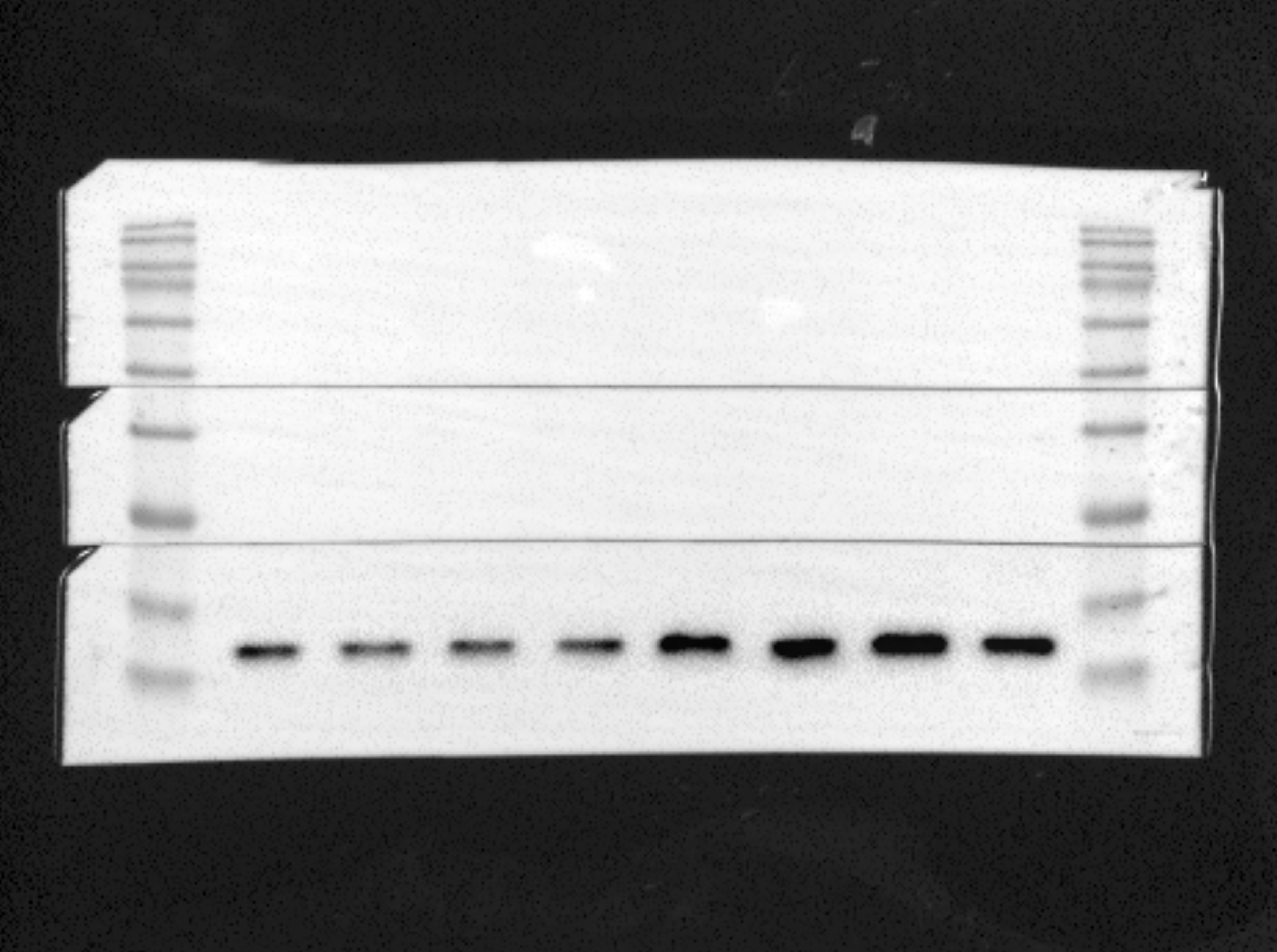

Supplement: Supplementary file 1 — Supplementary Material 1. [file 12958_2024_1250_MOESM1_ESM.zip › WB original picture/fig1-Nur77-p53-p21-p16-H2AX/H2AX/H2AX.tif]

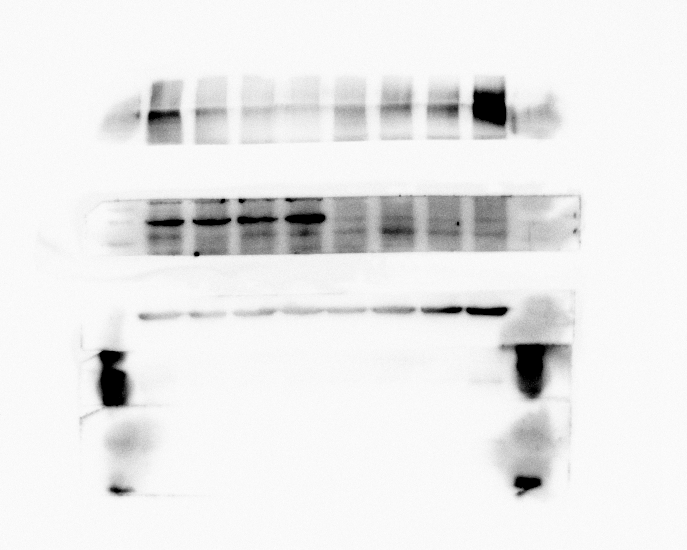

Supplement: Supplementary file 1 — Supplementary Material 1. [file 12958_2024_1250_MOESM1_ESM.zip › WB original picture/fig1-Nur77-p53-p21-p16-H2AX/NUR77/Nur77sample pattern.tif]

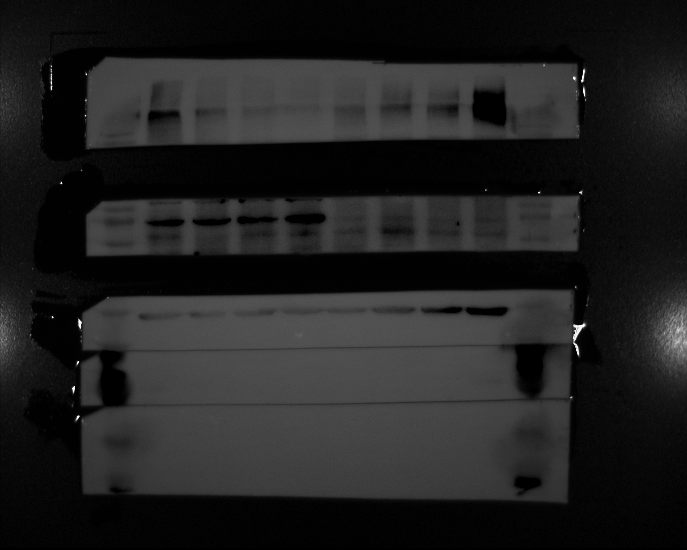

Supplement: Supplementary file 1 — Supplementary Material 1. [file 12958_2024_1250_MOESM1_ESM.zip › WB original picture/fig1-Nur77-p53-p21-p16-H2AX/NUR77/Nur77superposed graph.tif]

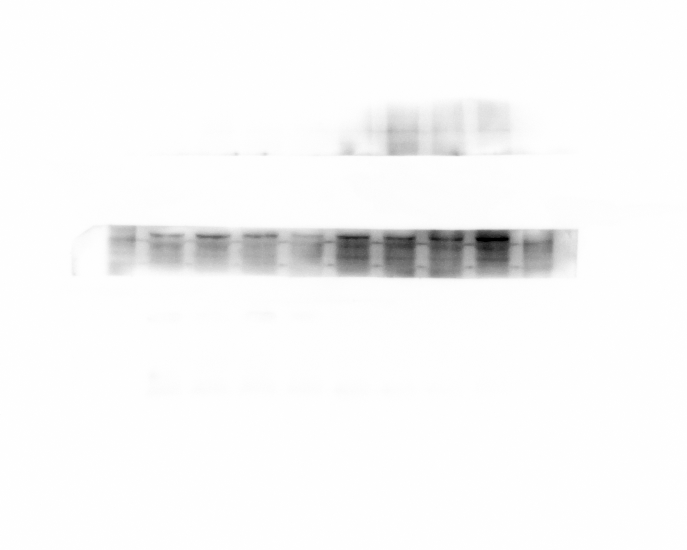

Supplement: Supplementary file 1 — Supplementary Material 1. [file 12958_2024_1250_MOESM1_ESM.zip › WB original picture/fig1-Nur77-p53-p21-p16-H2AX/P53/p53 sample pattern.tif]

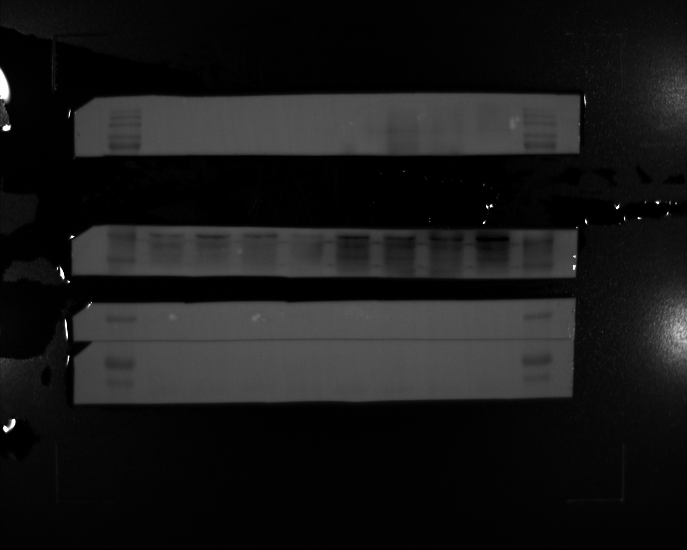

Supplement: Supplementary file 1 — Supplementary Material 1. [file 12958_2024_1250_MOESM1_ESM.zip › WB original picture/fig1-Nur77-p53-p21-p16-H2AX/P53/p53 superposed graph.tif]

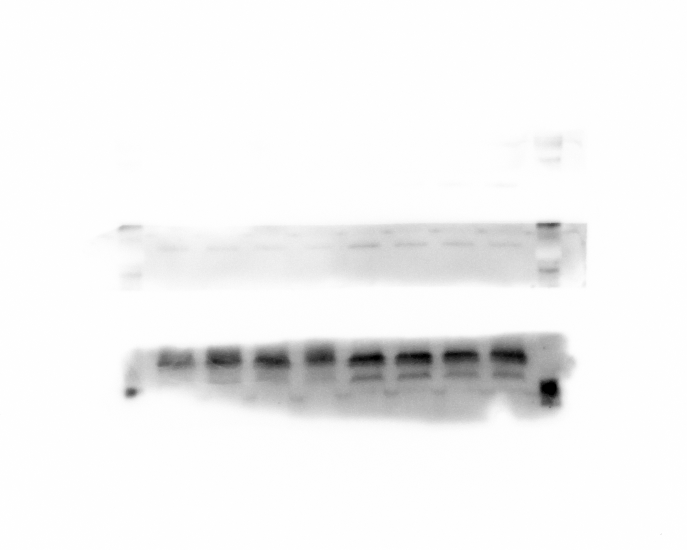

Supplement: Supplementary file 1 — Supplementary Material 1. [file 12958_2024_1250_MOESM1_ESM.zip › WB original picture/fig1-Nur77-p53-p21-p16-H2AX/p16/p16 sample pattern.tif]

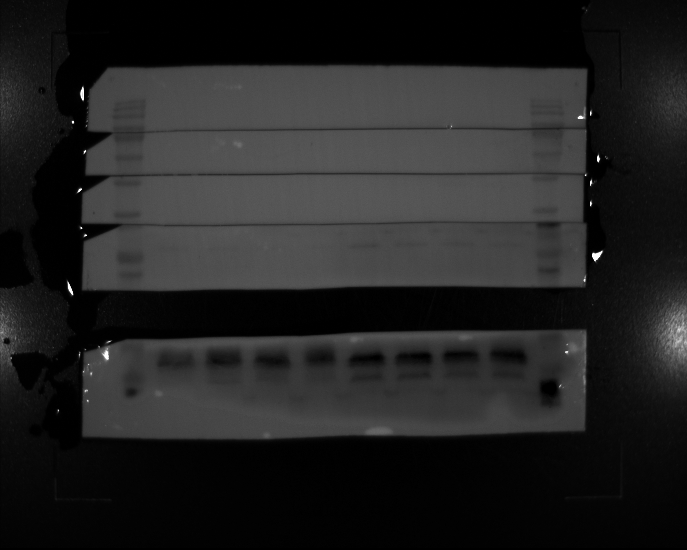

Supplement: Supplementary file 1 — Supplementary Material 1. [file 12958_2024_1250_MOESM1_ESM.zip › WB original picture/fig1-Nur77-p53-p21-p16-H2AX/p16/p16 superposed graph.tif]

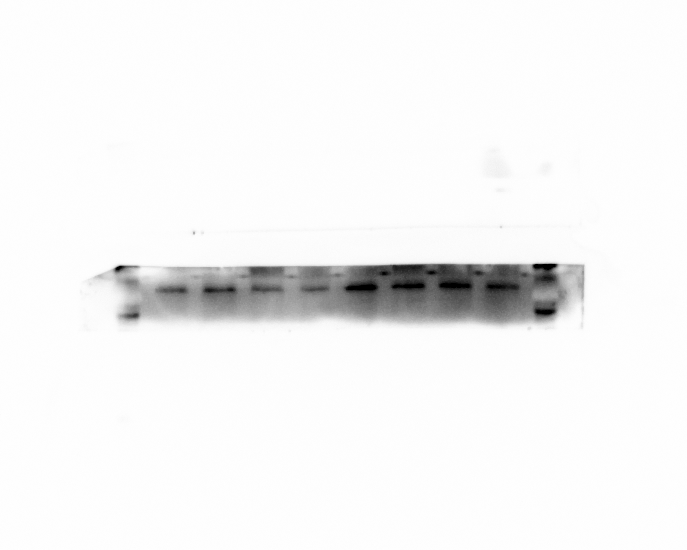

Supplement: Supplementary file 1 — Supplementary Material 1. [file 12958_2024_1250_MOESM1_ESM.zip › WB original picture/fig1-Nur77-p53-p21-p16-H2AX/p21/p21 sample pattern.tif]

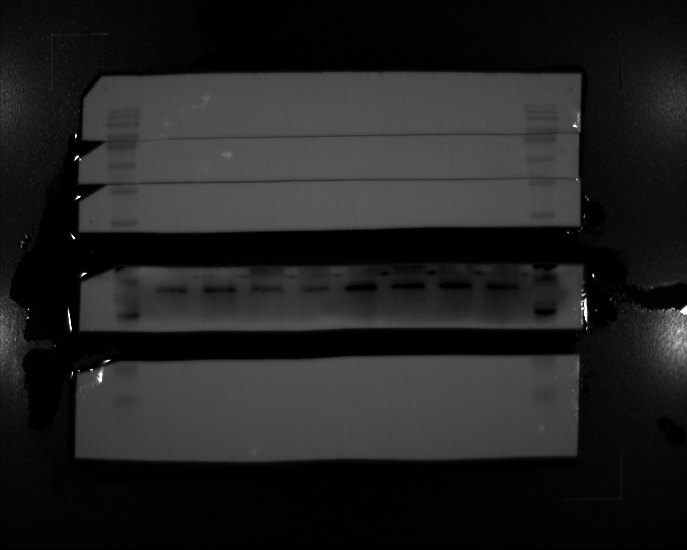

Supplement: Supplementary file 1 — Supplementary Material 1. [file 12958_2024_1250_MOESM1_ESM.zip › WB original picture/fig1-Nur77-p53-p21-p16-H2AX/p21/p21 superposed graph.tif]

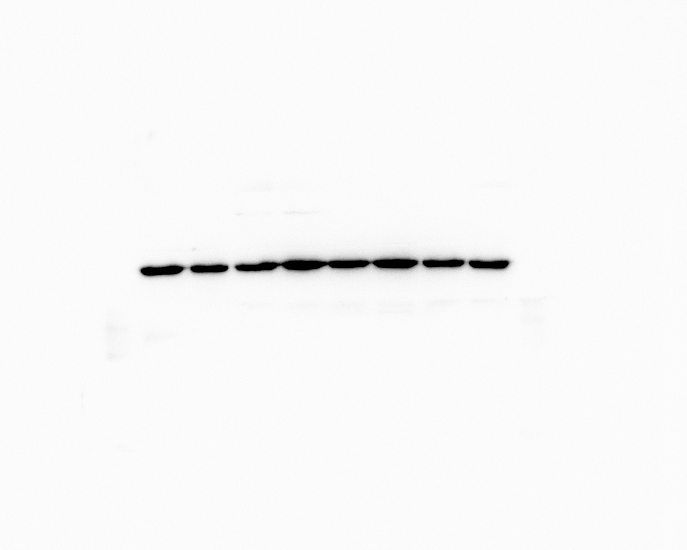

Supplement: Supplementary file 1 — Supplementary Material 1. [file 12958_2024_1250_MOESM1_ESM.zip › WB original picture/fig2-PINK1-Parkin-p62-LC3-GAPDH/Actin/Actin sample pattern.tif]

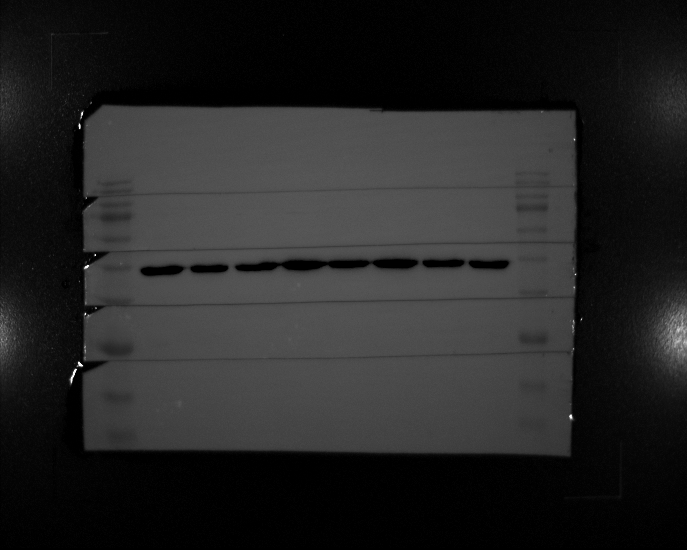

Supplement: Supplementary file 1 — Supplementary Material 1. [file 12958_2024_1250_MOESM1_ESM.zip › WB original picture/fig2-PINK1-Parkin-p62-LC3-GAPDH/Actin/Actin superposed graph.tif]

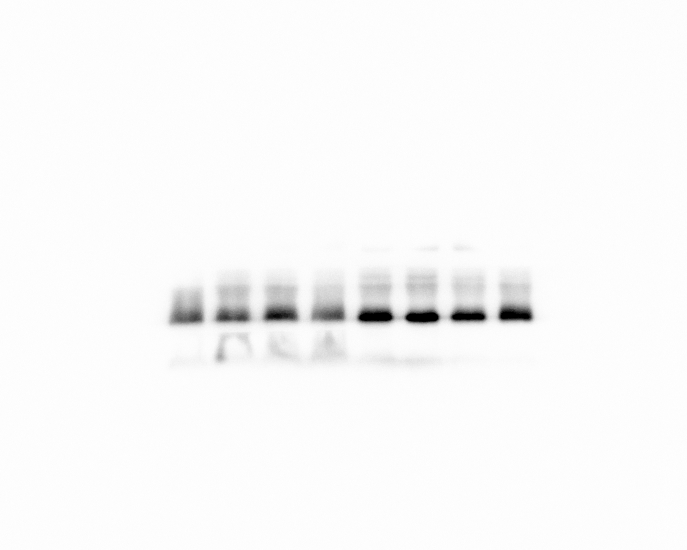

Supplement: Supplementary file 1 — Supplementary Material 1. [file 12958_2024_1250_MOESM1_ESM.zip › WB original picture/fig2-PINK1-Parkin-p62-LC3-GAPDH/BAX/Bax sample pattern.tif]

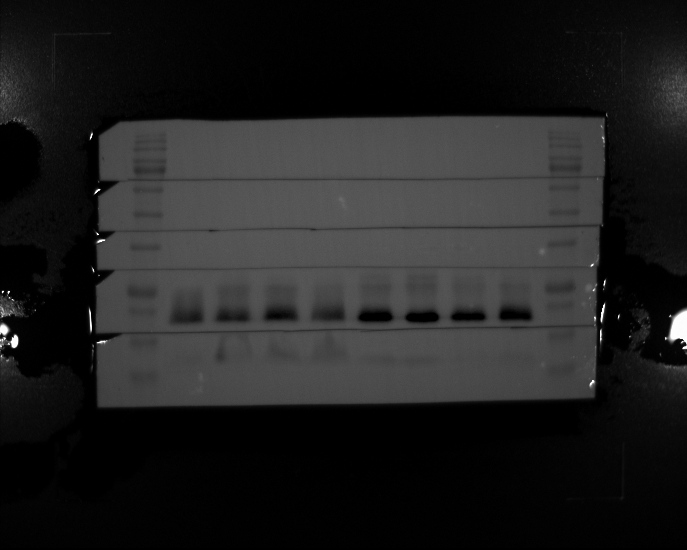

Supplement: Supplementary file 1 — Supplementary Material 1. [file 12958_2024_1250_MOESM1_ESM.zip › WB original picture/fig2-PINK1-Parkin-p62-LC3-GAPDH/BAX/Bax superposed graph.tif]

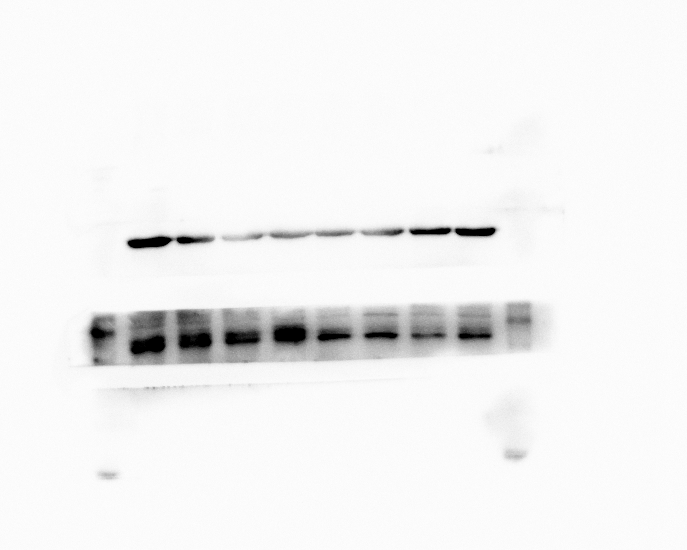

Supplement: Supplementary file 1 — Supplementary Material 1. [file 12958_2024_1250_MOESM1_ESM.zip › WB original picture/fig2-PINK1-Parkin-p62-LC3-GAPDH/Bcl2/Bcl2 sample pattern.tif]

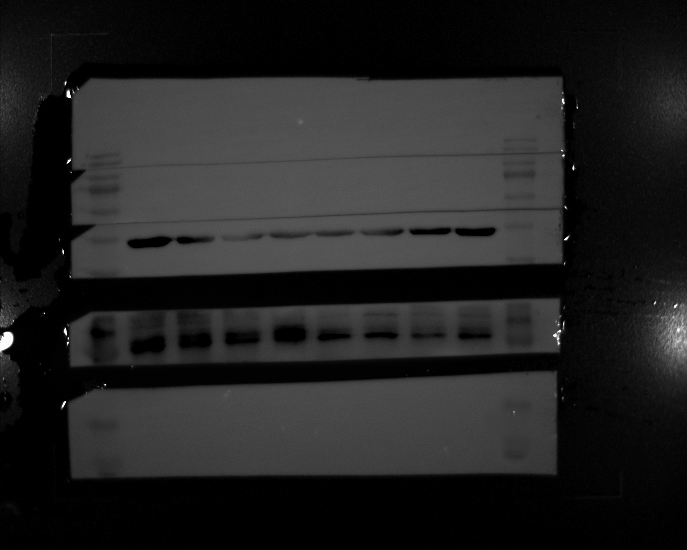

Supplement: Supplementary file 1 — Supplementary Material 1. [file 12958_2024_1250_MOESM1_ESM.zip › WB original picture/fig2-PINK1-Parkin-p62-LC3-GAPDH/Bcl2/Bcl2 superposed graph.tif]

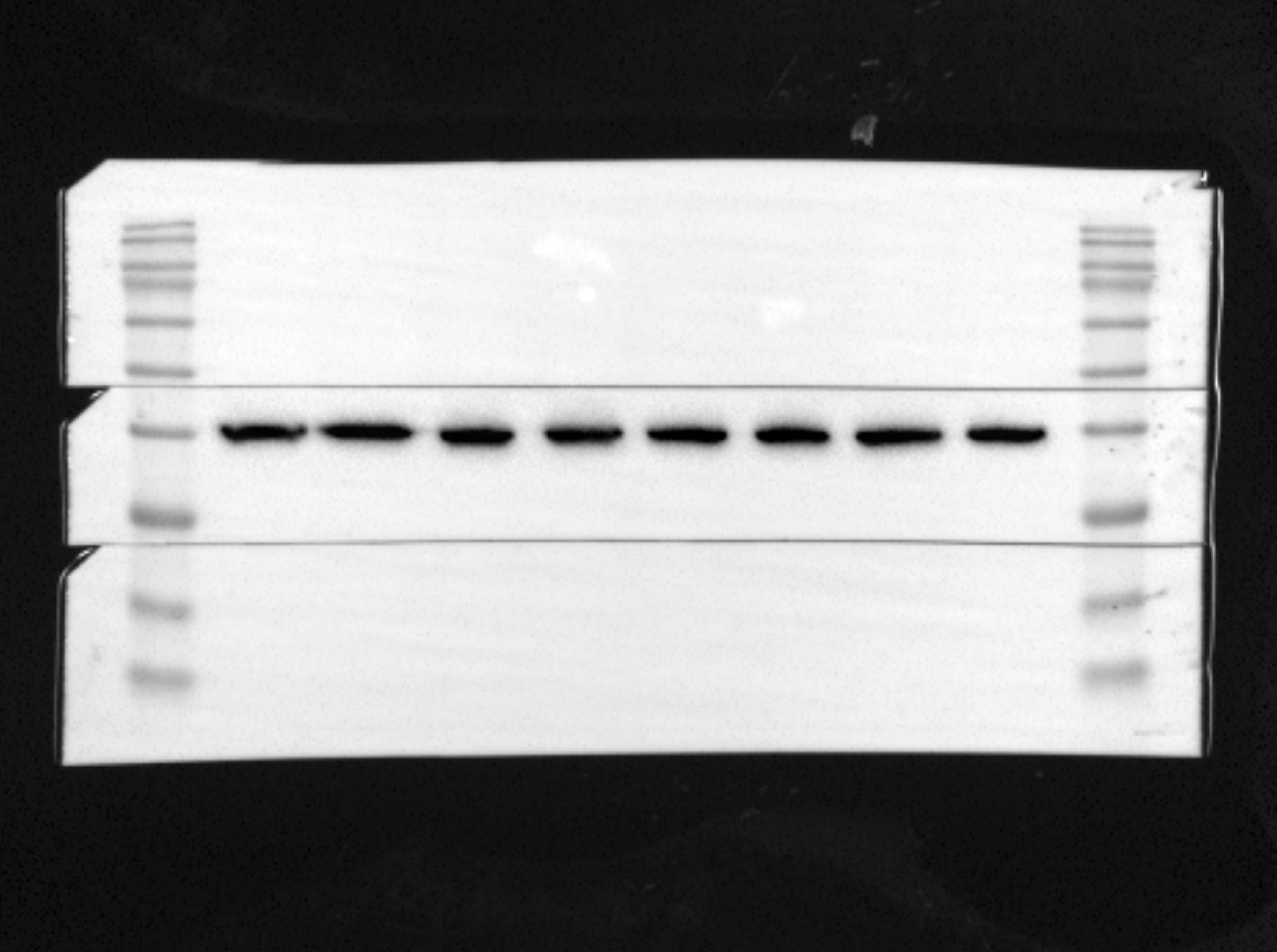

Supplement: Supplementary file 1 — Supplementary Material 1. [file 12958_2024_1250_MOESM1_ESM.zip › WB original picture/fig2-PINK1-Parkin-p62-LC3-GAPDH/GAPDH/GAPDH.tif]

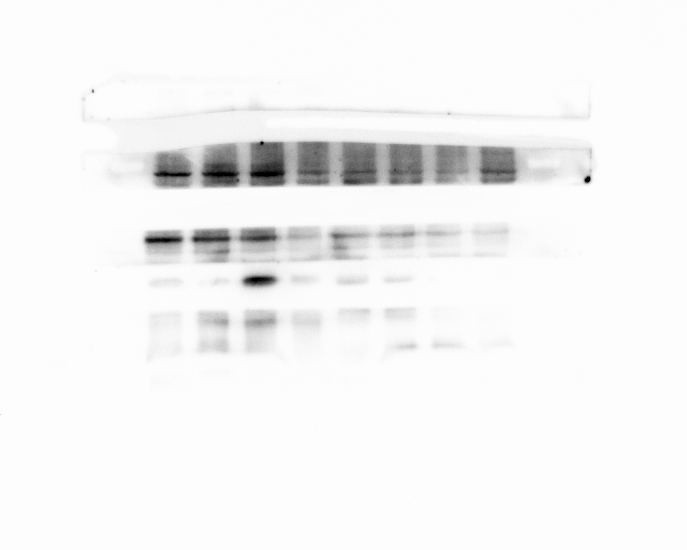

Supplement: Supplementary file 1 — Supplementary Material 1. [file 12958_2024_1250_MOESM1_ESM.zip › WB original picture/fig2-PINK1-Parkin-p62-LC3-GAPDH/PINK1/PINK1 sample pattern.tif]

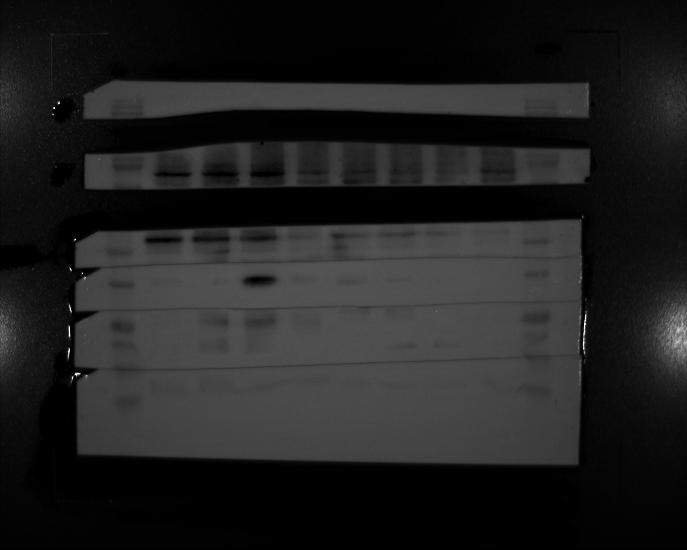

Supplement: Supplementary file 1 — Supplementary Material 1. [file 12958_2024_1250_MOESM1_ESM.zip › WB original picture/fig2-PINK1-Parkin-p62-LC3-GAPDH/PINK1/PINK1 superposed graph.tif]

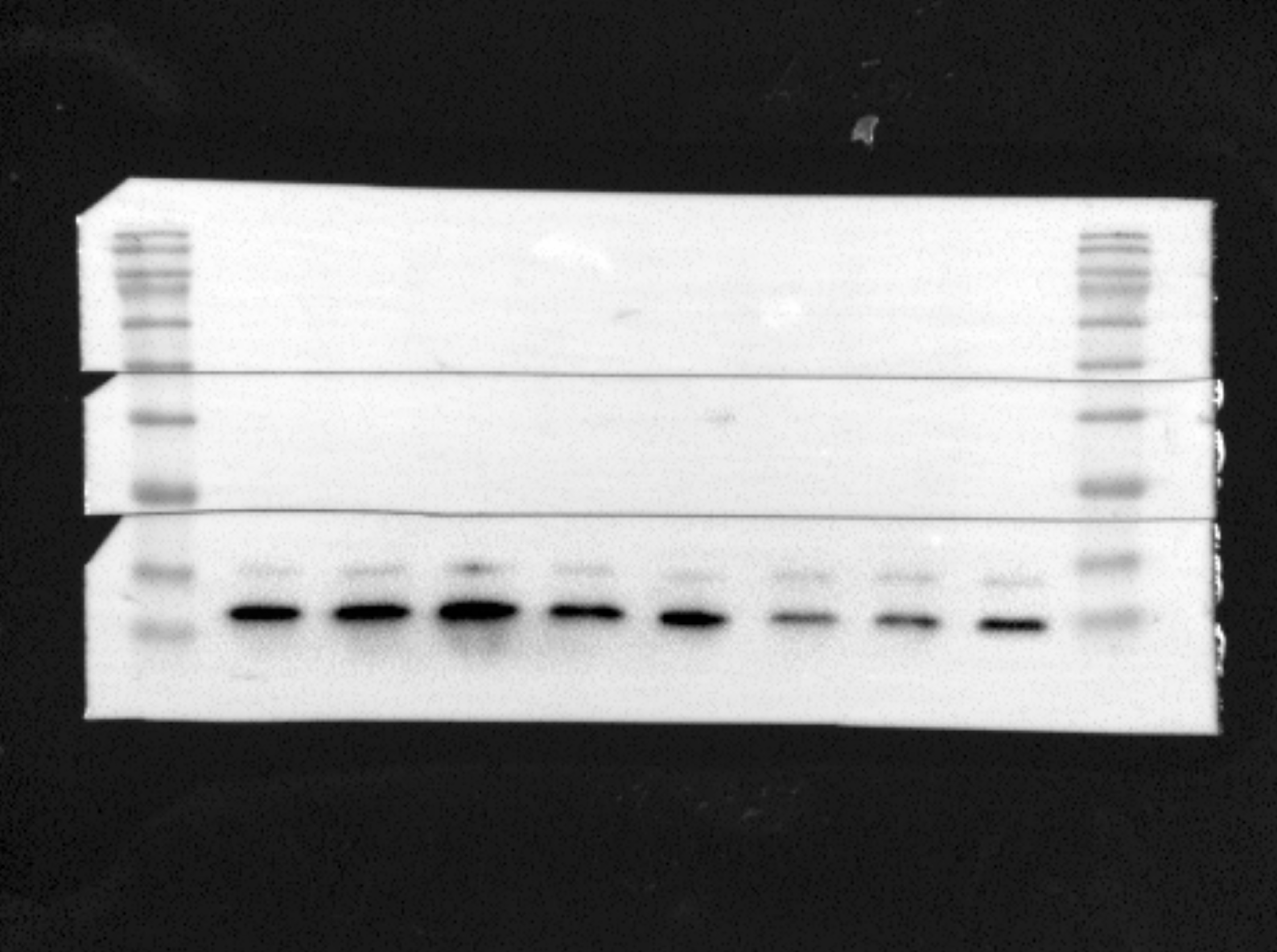

Supplement: Supplementary file 1 — Supplementary Material 1. [file 12958_2024_1250_MOESM1_ESM.zip › WB original picture/fig2-PINK1-Parkin-p62-LC3-GAPDH/lc3/LC3.tif]

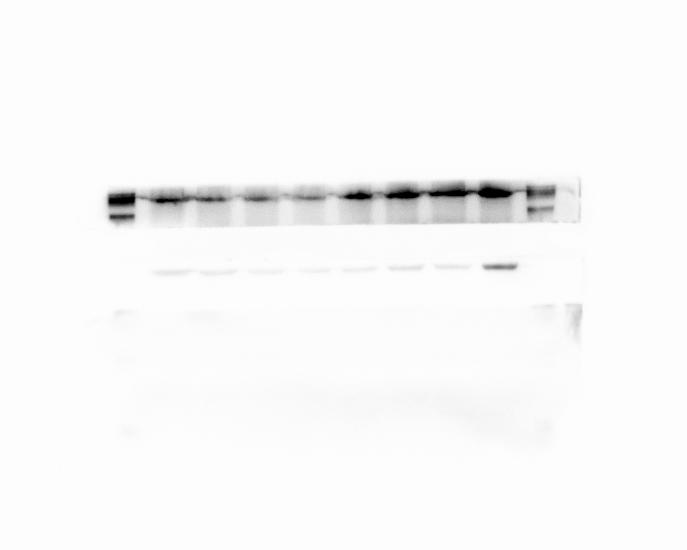

Supplement: Supplementary file 1 — Supplementary Material 1. [file 12958_2024_1250_MOESM1_ESM.zip › WB original picture/fig2-PINK1-Parkin-p62-LC3-GAPDH/p62/p62 sample pattern.tif]

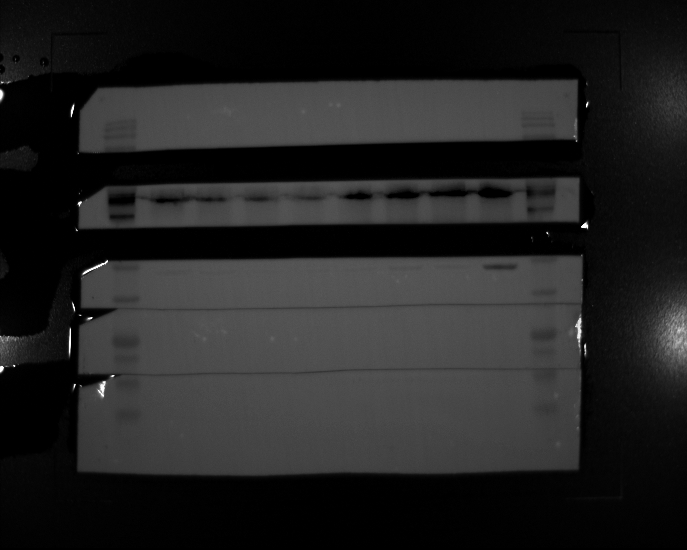

Supplement: Supplementary file 1 — Supplementary Material 1. [file 12958_2024_1250_MOESM1_ESM.zip › WB original picture/fig2-PINK1-Parkin-p62-LC3-GAPDH/p62/p62 superposed graph.tif]

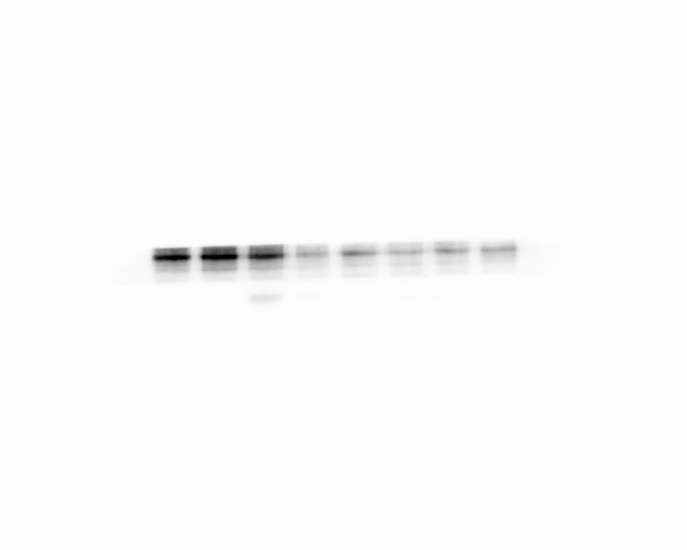

Supplement: Supplementary file 1 — Supplementary Material 1. [file 12958_2024_1250_MOESM1_ESM.zip › WB original picture/fig2-PINK1-Parkin-p62-LC3-GAPDH/parkin/parkin sample pattern.tif]

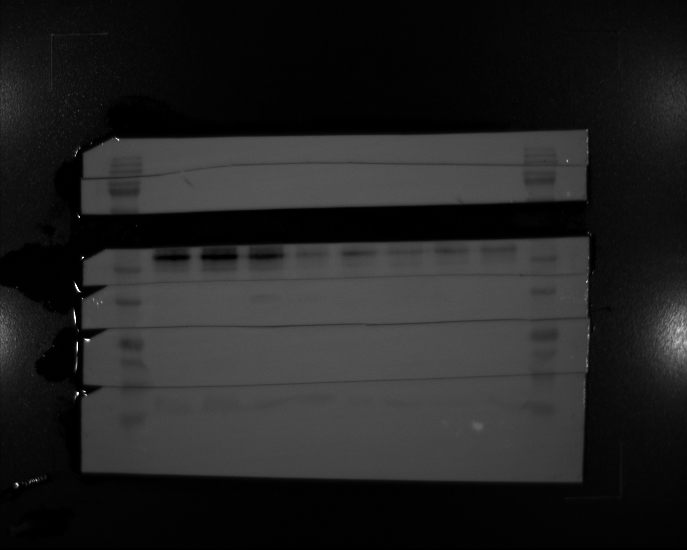

Supplement: Supplementary file 1 — Supplementary Material 1. [file 12958_2024_1250_MOESM1_ESM.zip › WB original picture/fig2-PINK1-Parkin-p62-LC3-GAPDH/parkin/parkin superposed graph.tif]

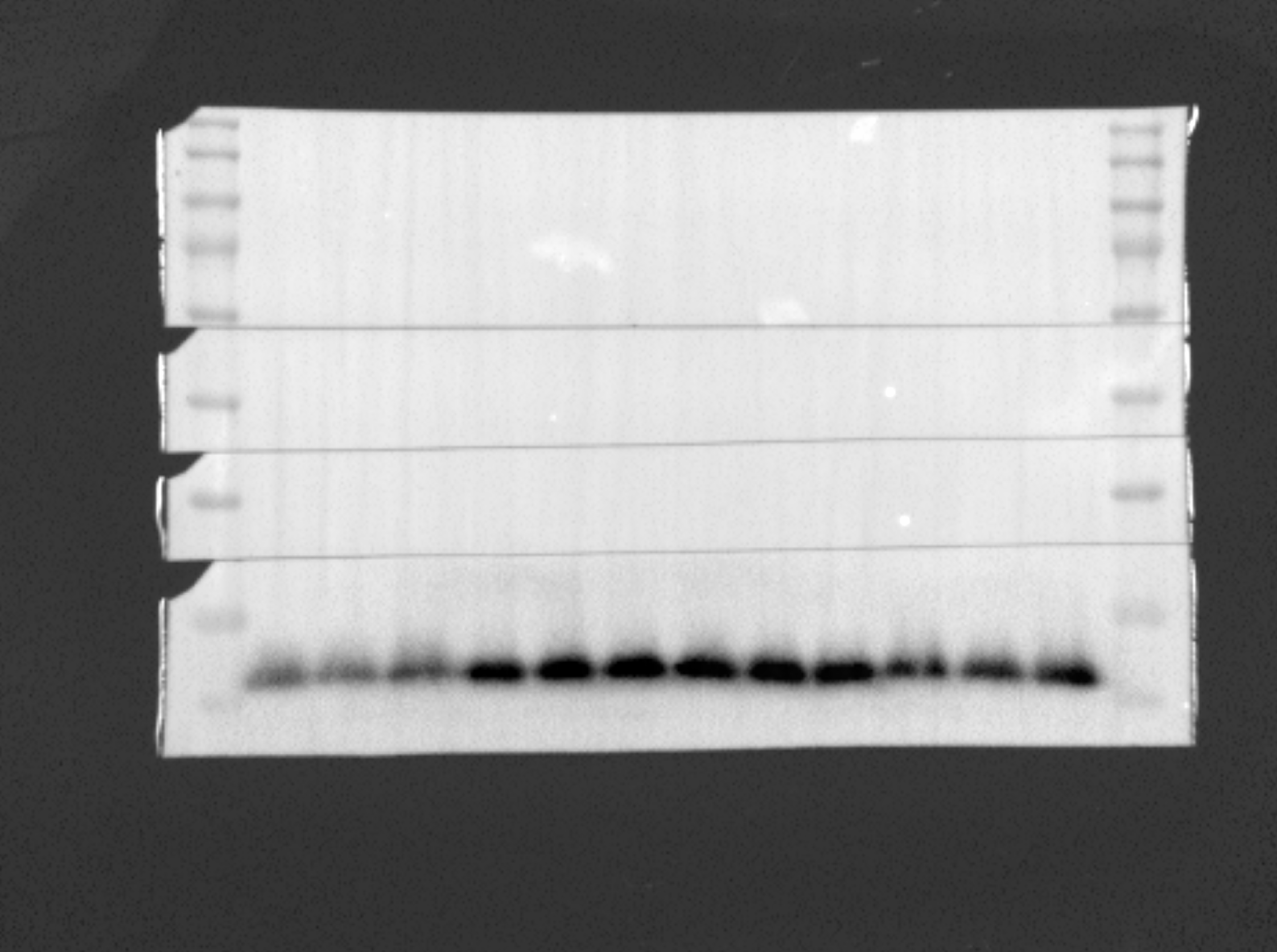

Supplement: Supplementary file 1 — Supplementary Material 1. [file 12958_2024_1250_MOESM1_ESM.zip › WB original picture/fig5-Bcl2-BAX/Bax/Bax.tif]

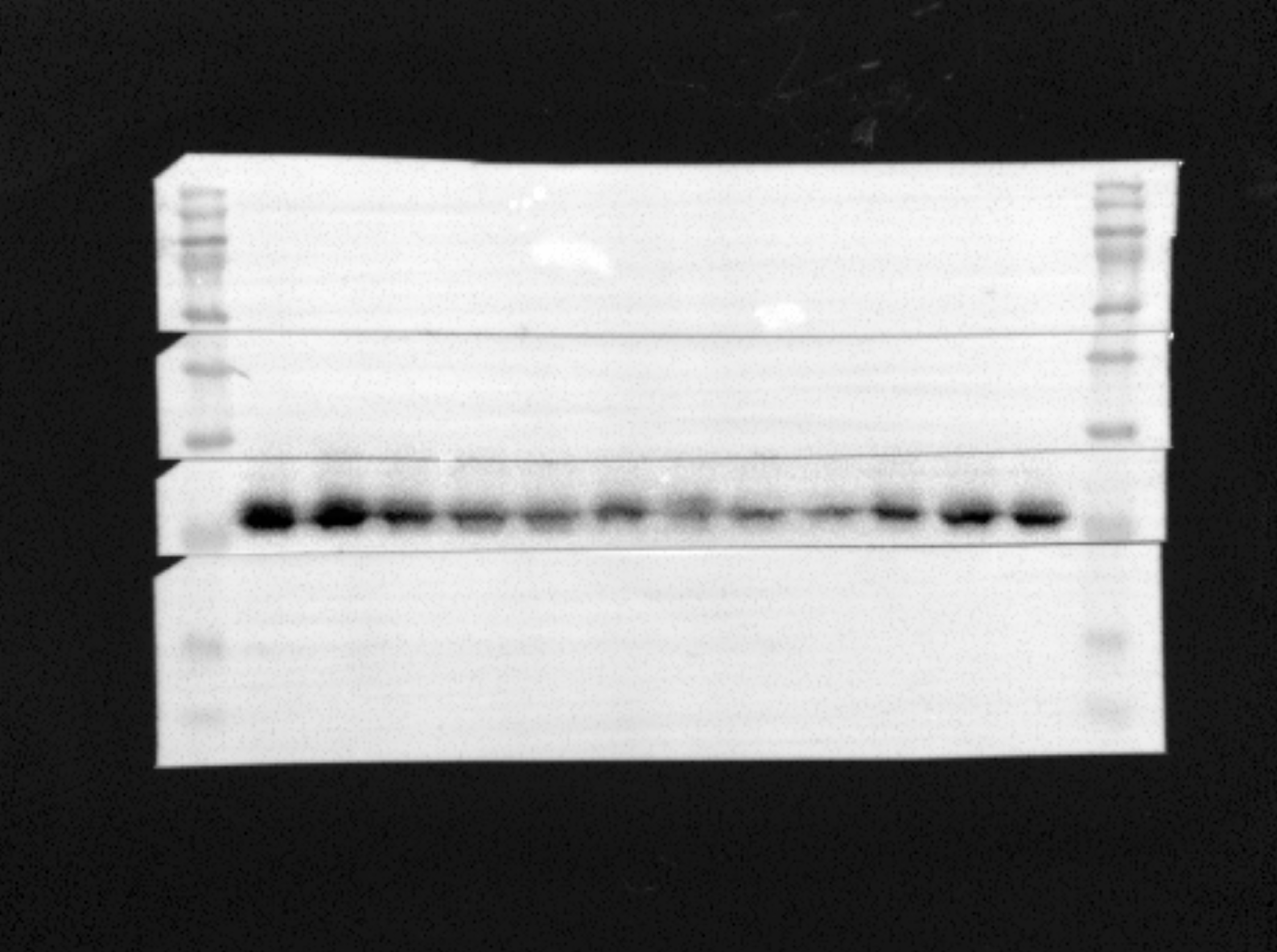

Supplement: Supplementary file 1 — Supplementary Material 1. [file 12958_2024_1250_MOESM1_ESM.zip › WB original picture/fig5-Bcl2-BAX/Bcl2/Bcl2.tif]

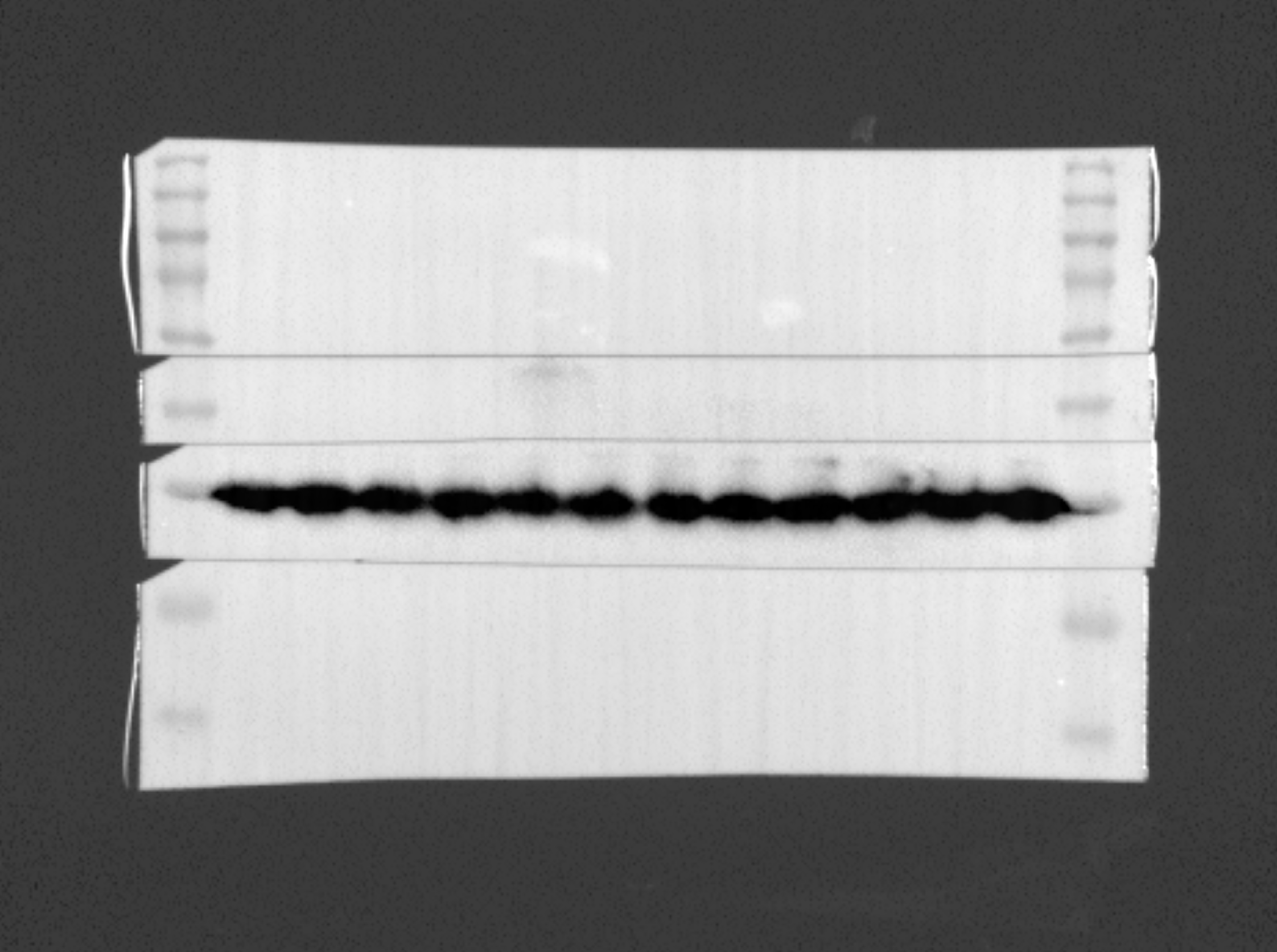

Supplement: Supplementary file 1 — Supplementary Material 1. [file 12958_2024_1250_MOESM1_ESM.zip › WB original picture/fig5-Bcl2-BAX/GAPDH/GAPDH.tif]

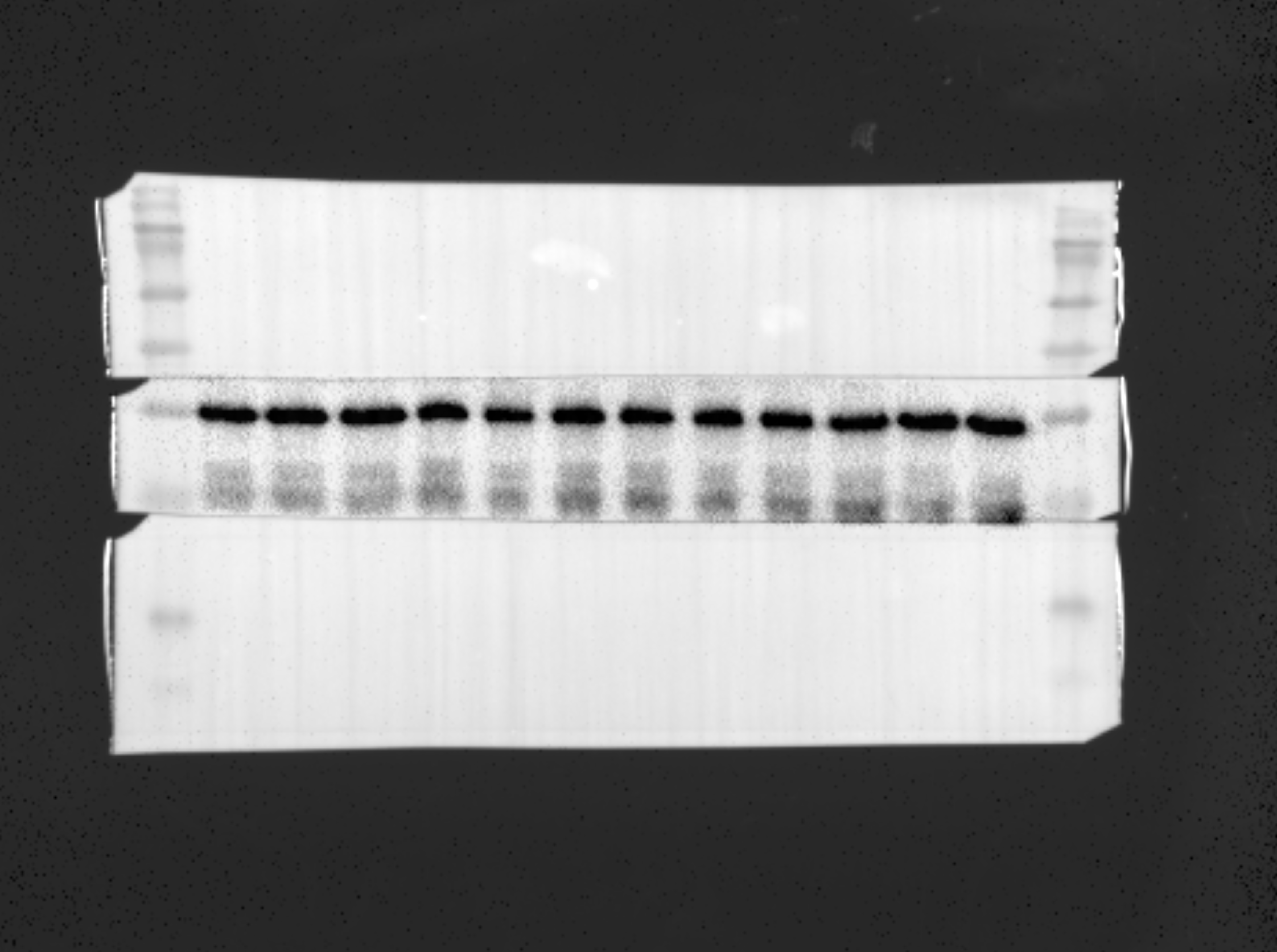

Supplement: Supplementary file 1 — Supplementary Material 1. [file 12958_2024_1250_MOESM1_ESM.zip › WB original picture/fig6-Nur77-p53-p21-p16-H2AX/GAPDH/GAPDH.tif]

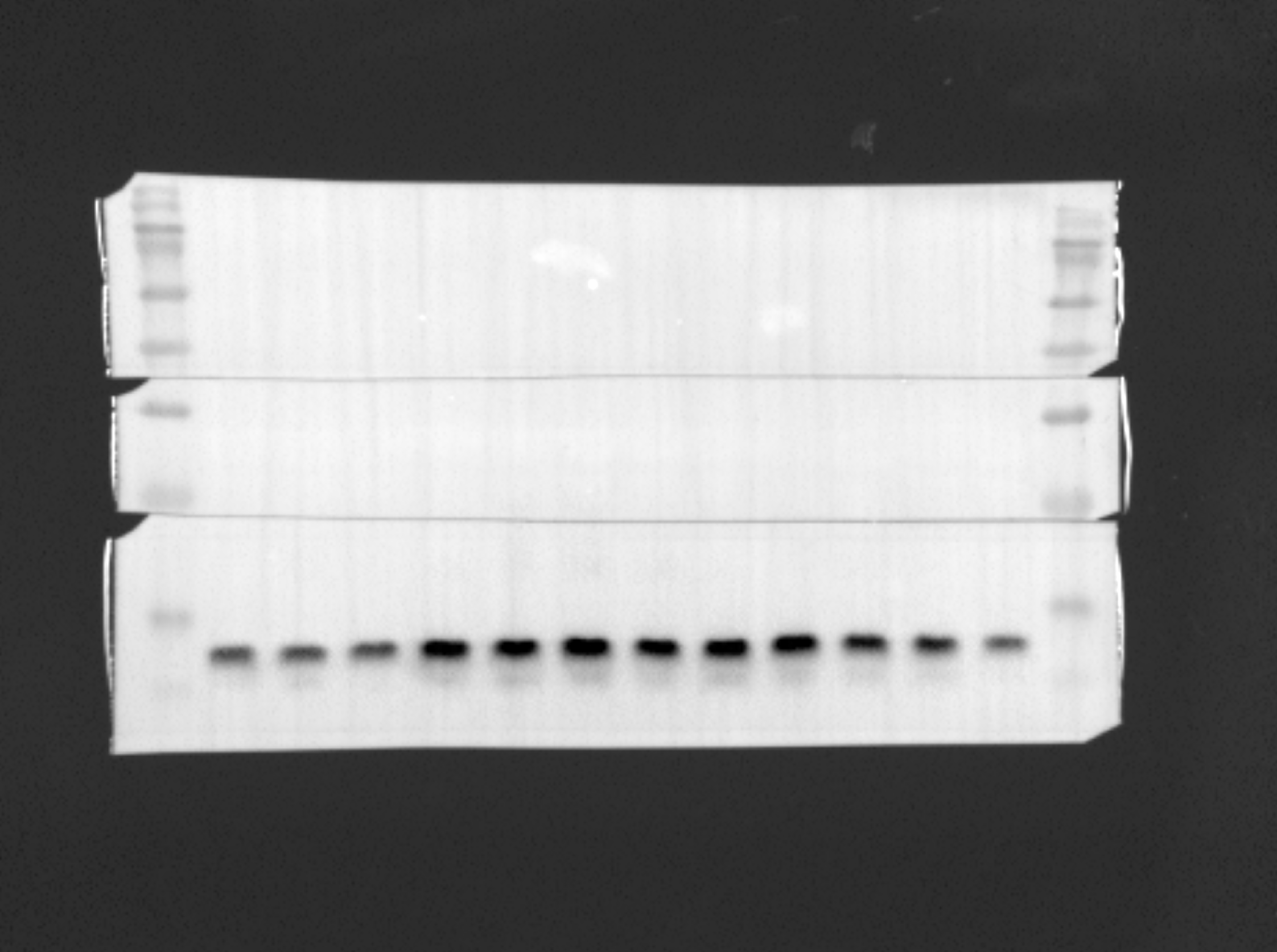

Supplement: Supplementary file 1 — Supplementary Material 1. [file 12958_2024_1250_MOESM1_ESM.zip › WB original picture/fig6-Nur77-p53-p21-p16-H2AX/H2AX/H2AX.tif]

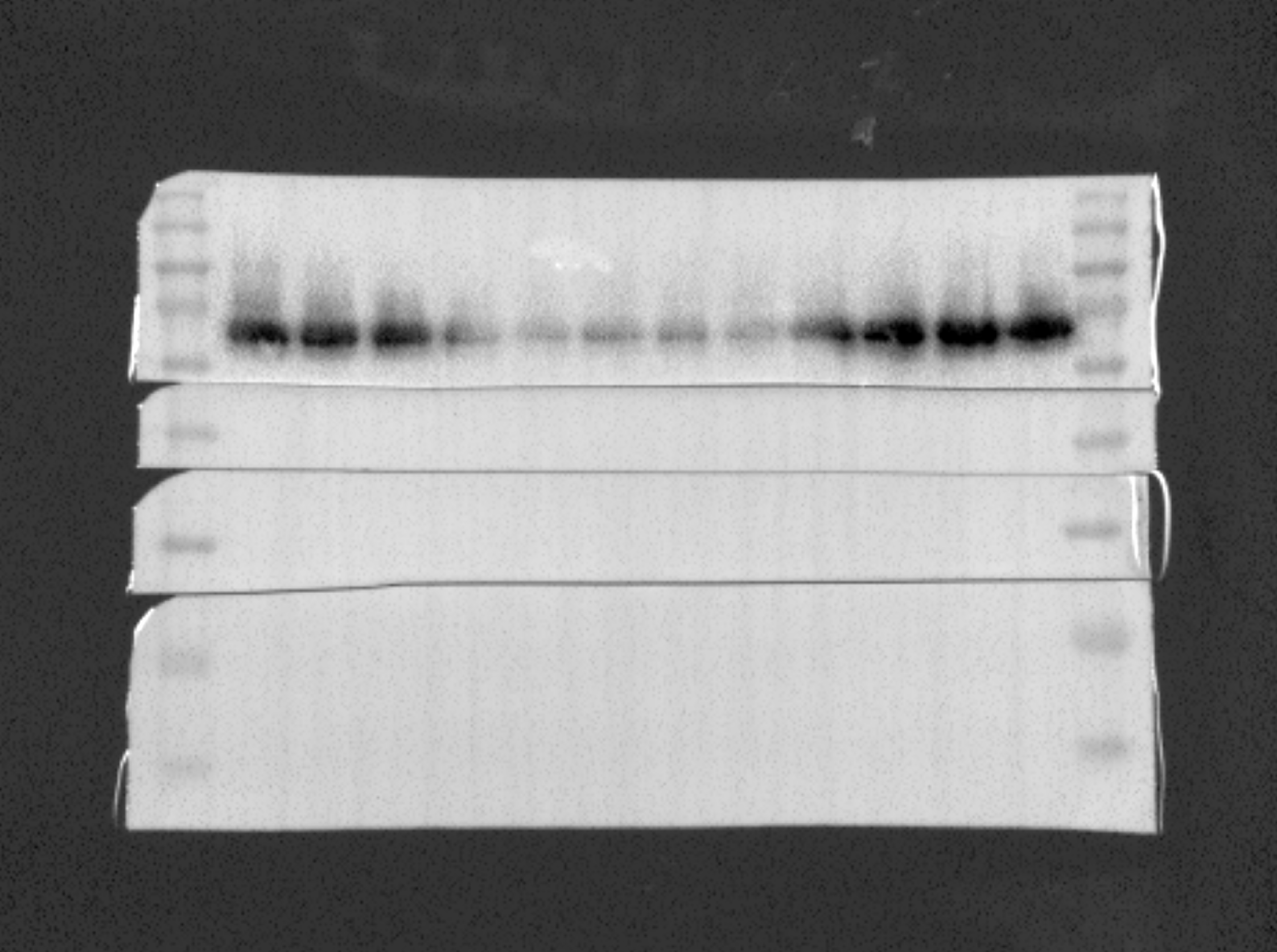

Supplement: Supplementary file 1 — Supplementary Material 1. [file 12958_2024_1250_MOESM1_ESM.zip › WB original picture/fig6-Nur77-p53-p21-p16-H2AX/Nur77/Nur77.tif]

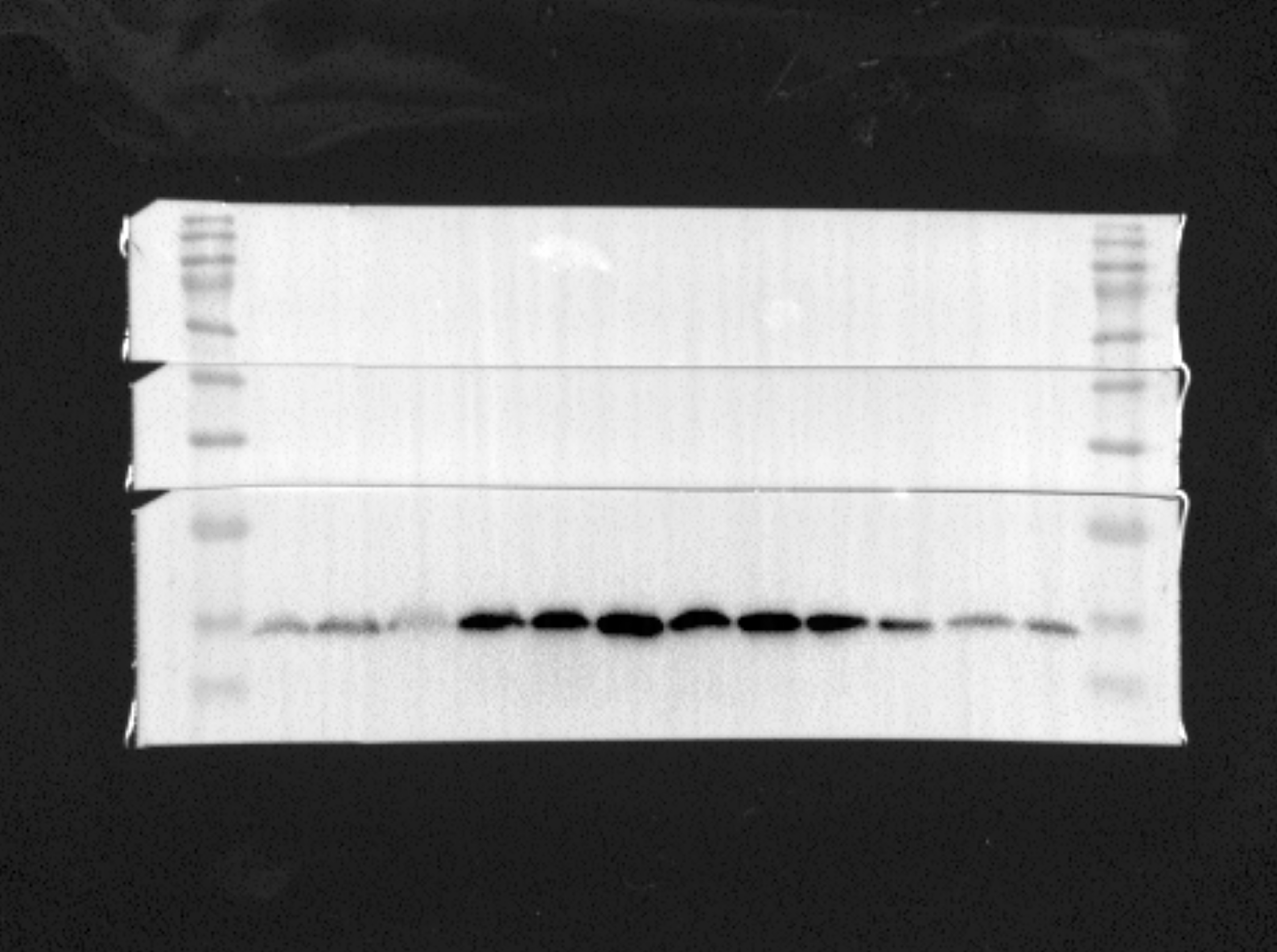

Supplement: Supplementary file 1 — Supplementary Material 1. [file 12958_2024_1250_MOESM1_ESM.zip › WB original picture/fig6-Nur77-p53-p21-p16-H2AX/p16/p16.tif]

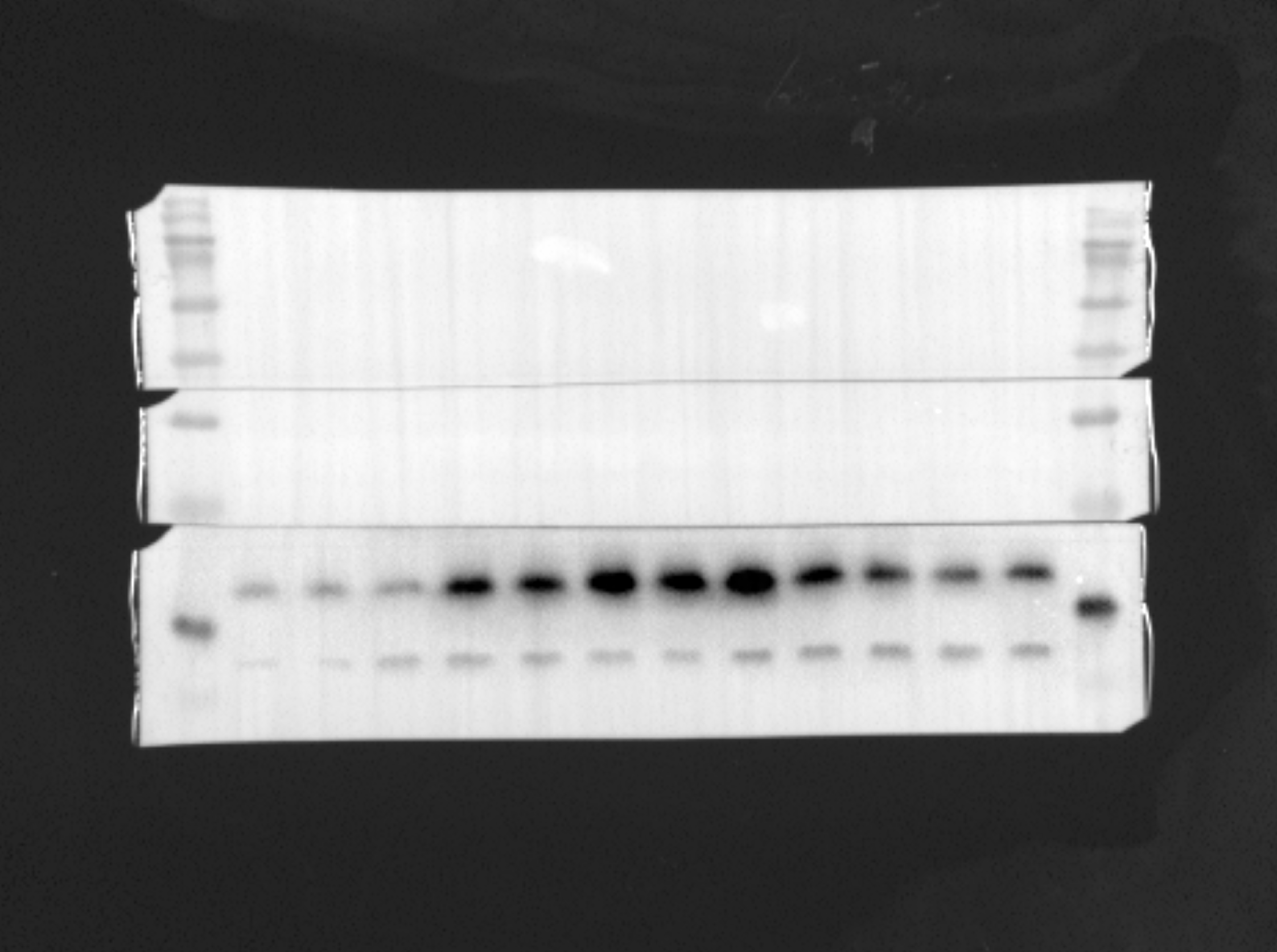

Supplement: Supplementary file 1 — Supplementary Material 1. [file 12958_2024_1250_MOESM1_ESM.zip › WB original picture/fig6-Nur77-p53-p21-p16-H2AX/p21/p21.tif]

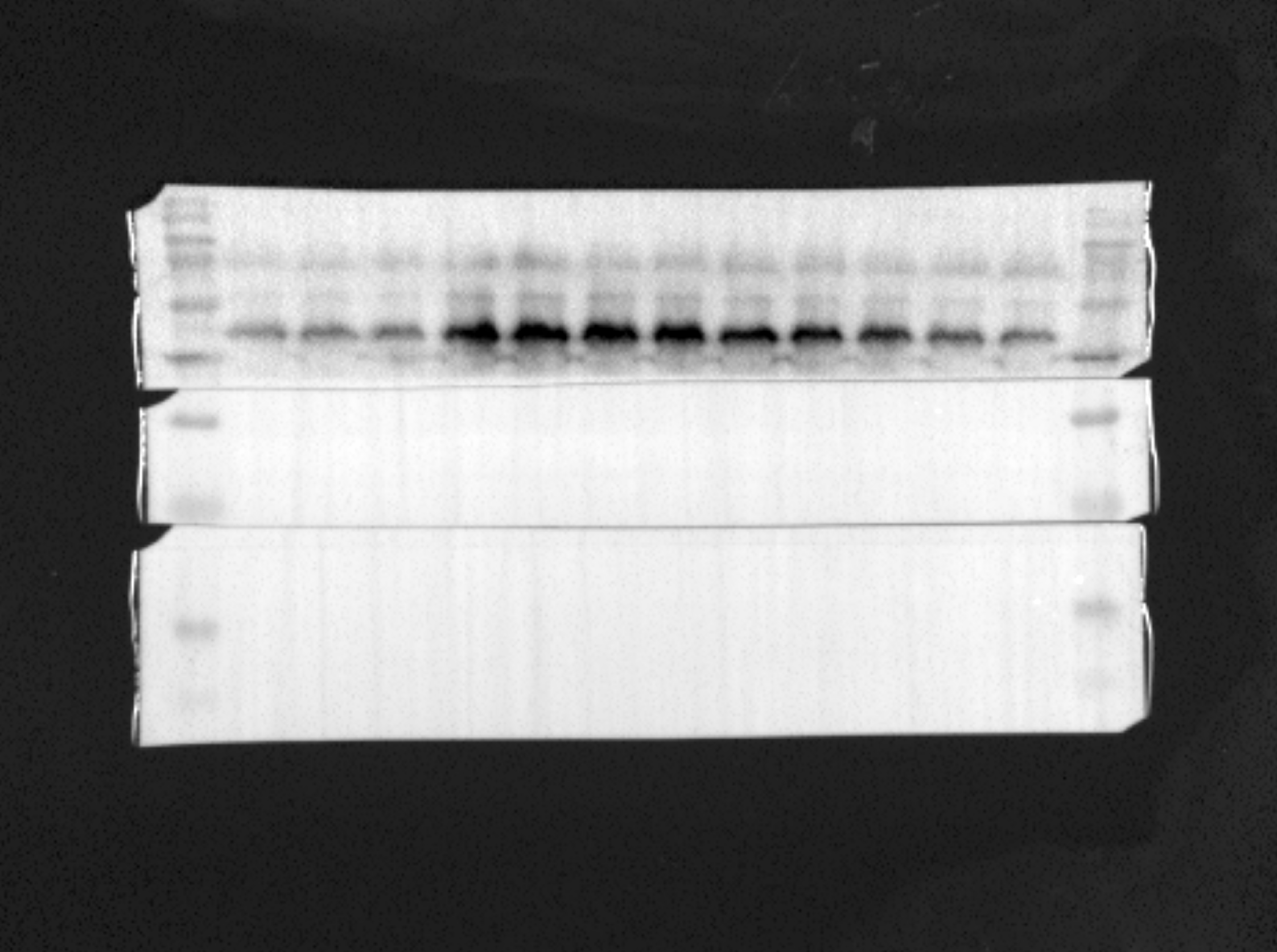

Supplement: Supplementary file 1 — Supplementary Material 1. [file 12958_2024_1250_MOESM1_ESM.zip › WB original picture/fig6-Nur77-p53-p21-p16-H2AX/p53/p53.tif]

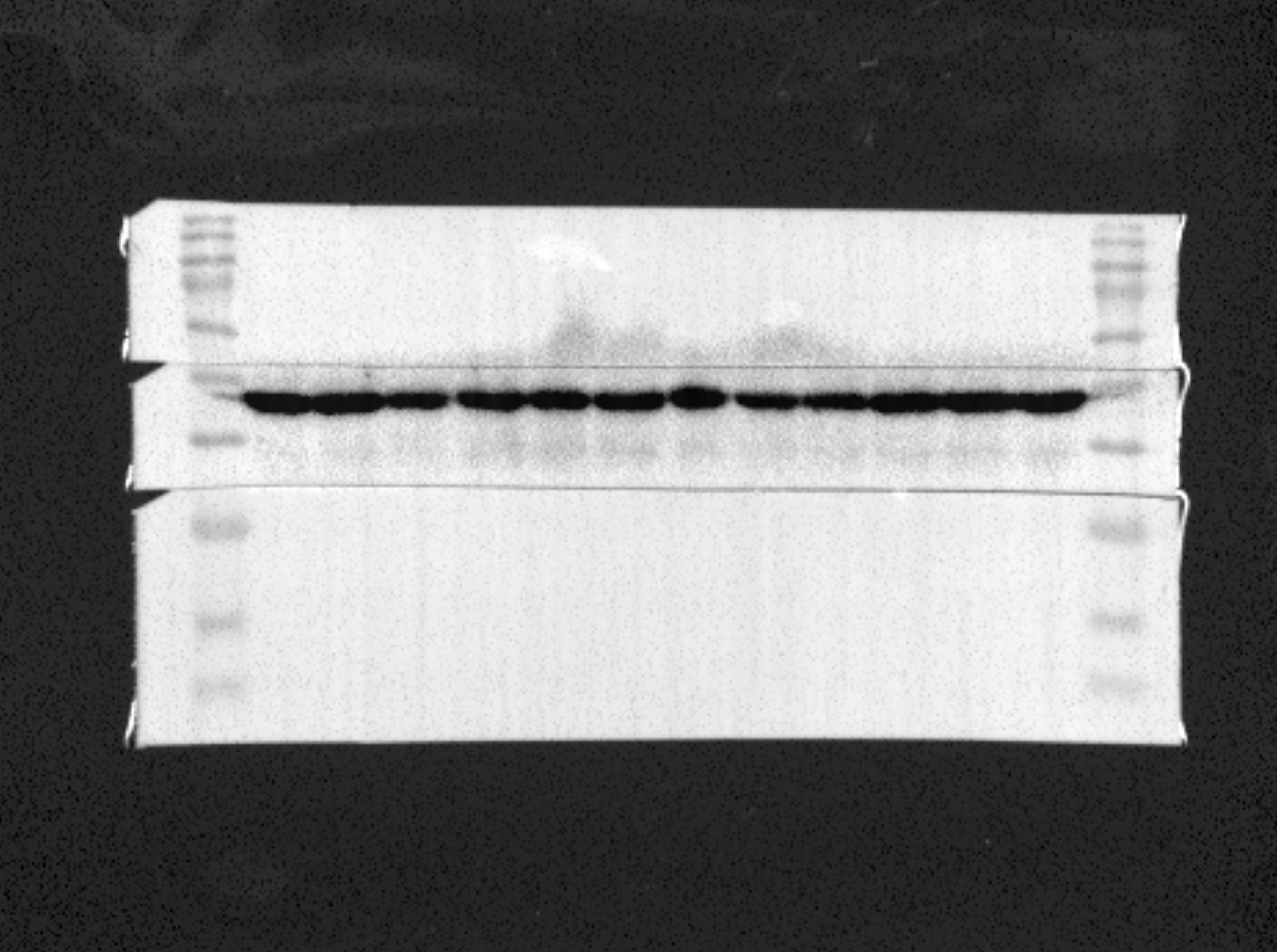

Supplement: Supplementary file 1 — Supplementary Material 1. [file 12958_2024_1250_MOESM1_ESM.zip › WB original picture/fig7-PINK1-parkin-p62-LC3-Actin/Actin/Actin.tif]

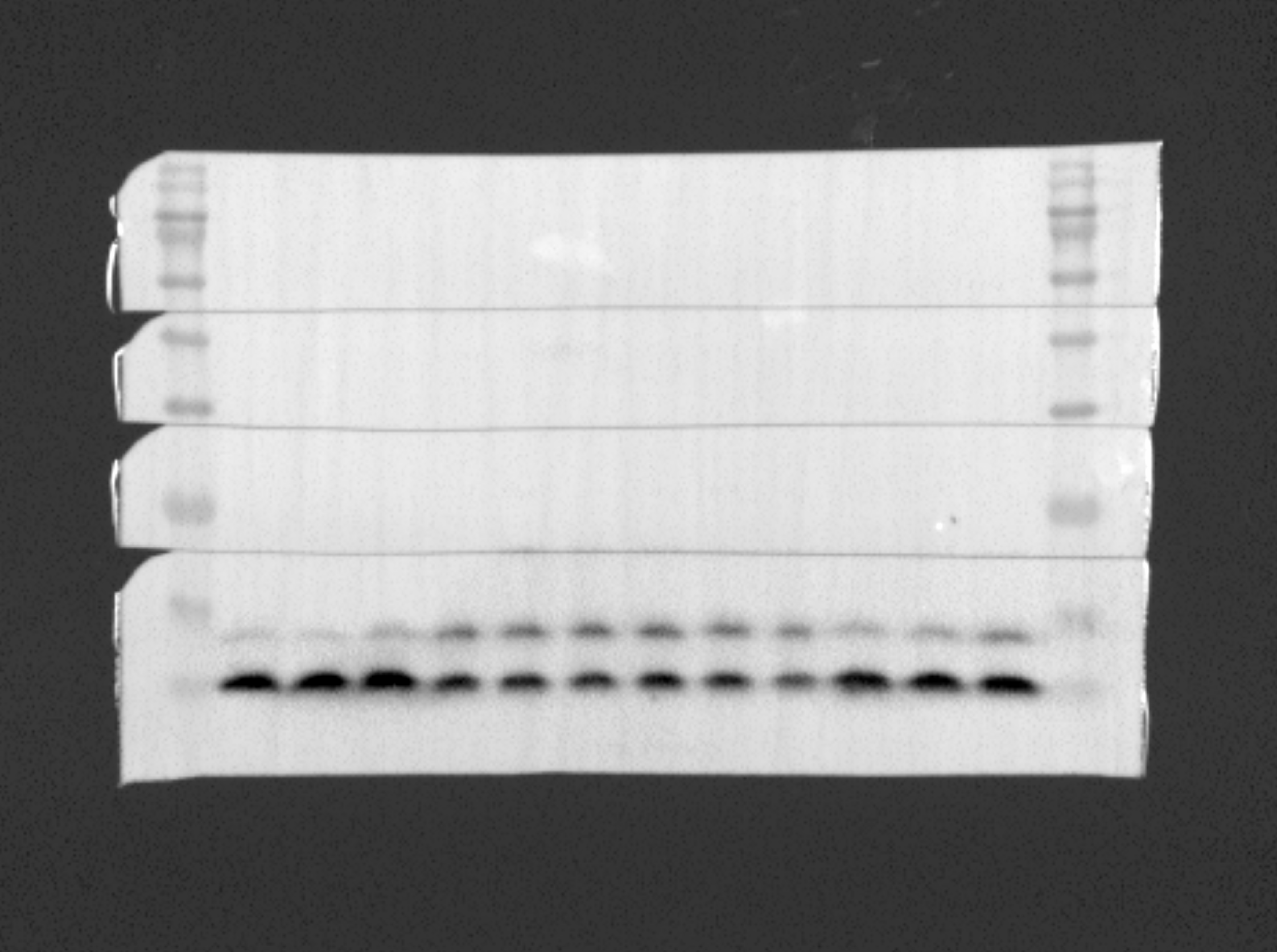

Supplement: Supplementary file 1 — Supplementary Material 1. [file 12958_2024_1250_MOESM1_ESM.zip › WB original picture/fig7-PINK1-parkin-p62-LC3-Actin/LC3/LC3.tif]

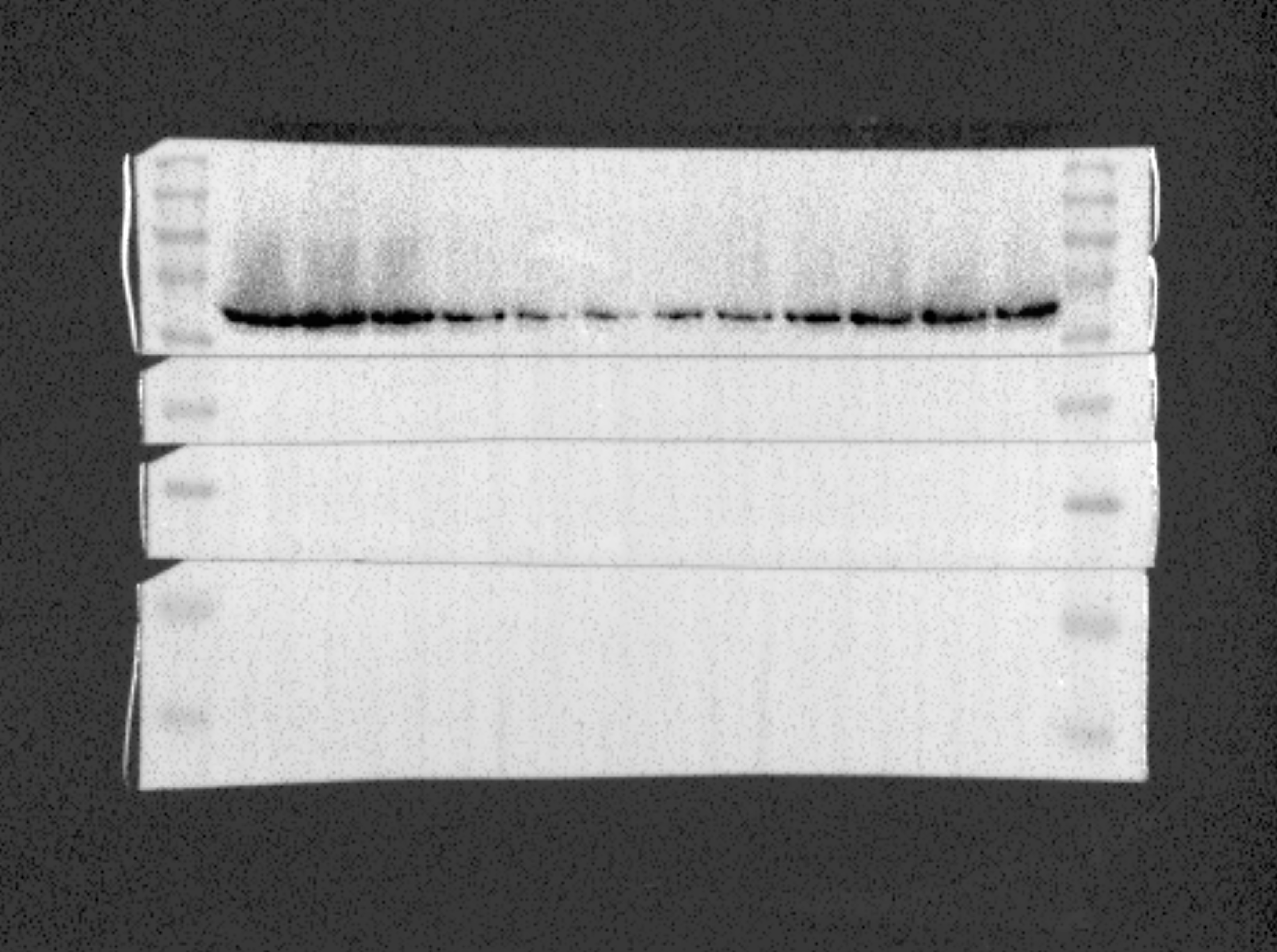

Supplement: Supplementary file 1 — Supplementary Material 1. [file 12958_2024_1250_MOESM1_ESM.zip › WB original picture/fig7-PINK1-parkin-p62-LC3-Actin/PINK1/PINK1.tif]

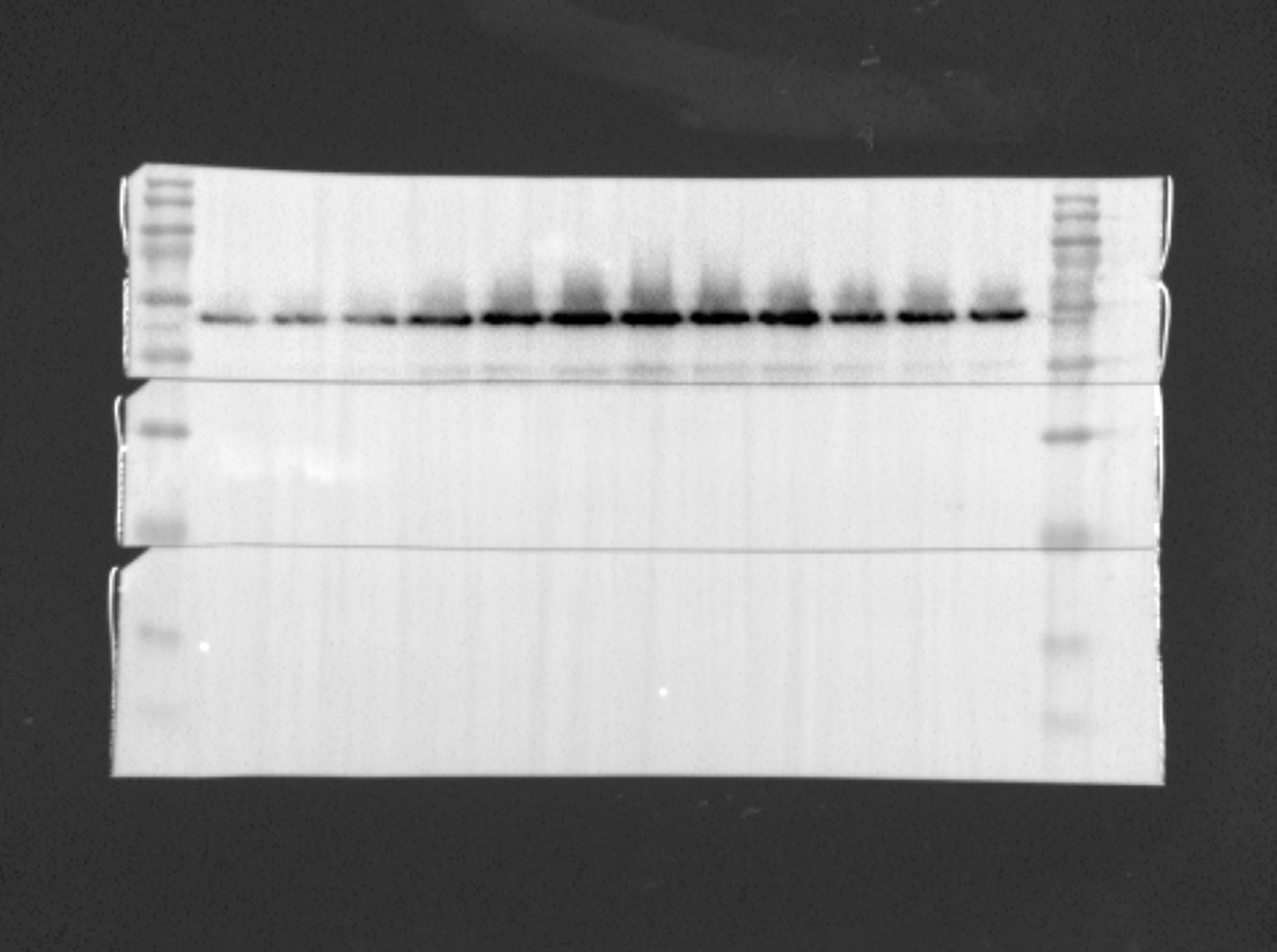

Supplement: Supplementary file 1 — Supplementary Material 1. [file 12958_2024_1250_MOESM1_ESM.zip › WB original picture/fig7-PINK1-parkin-p62-LC3-Actin/p62/p62.tif]

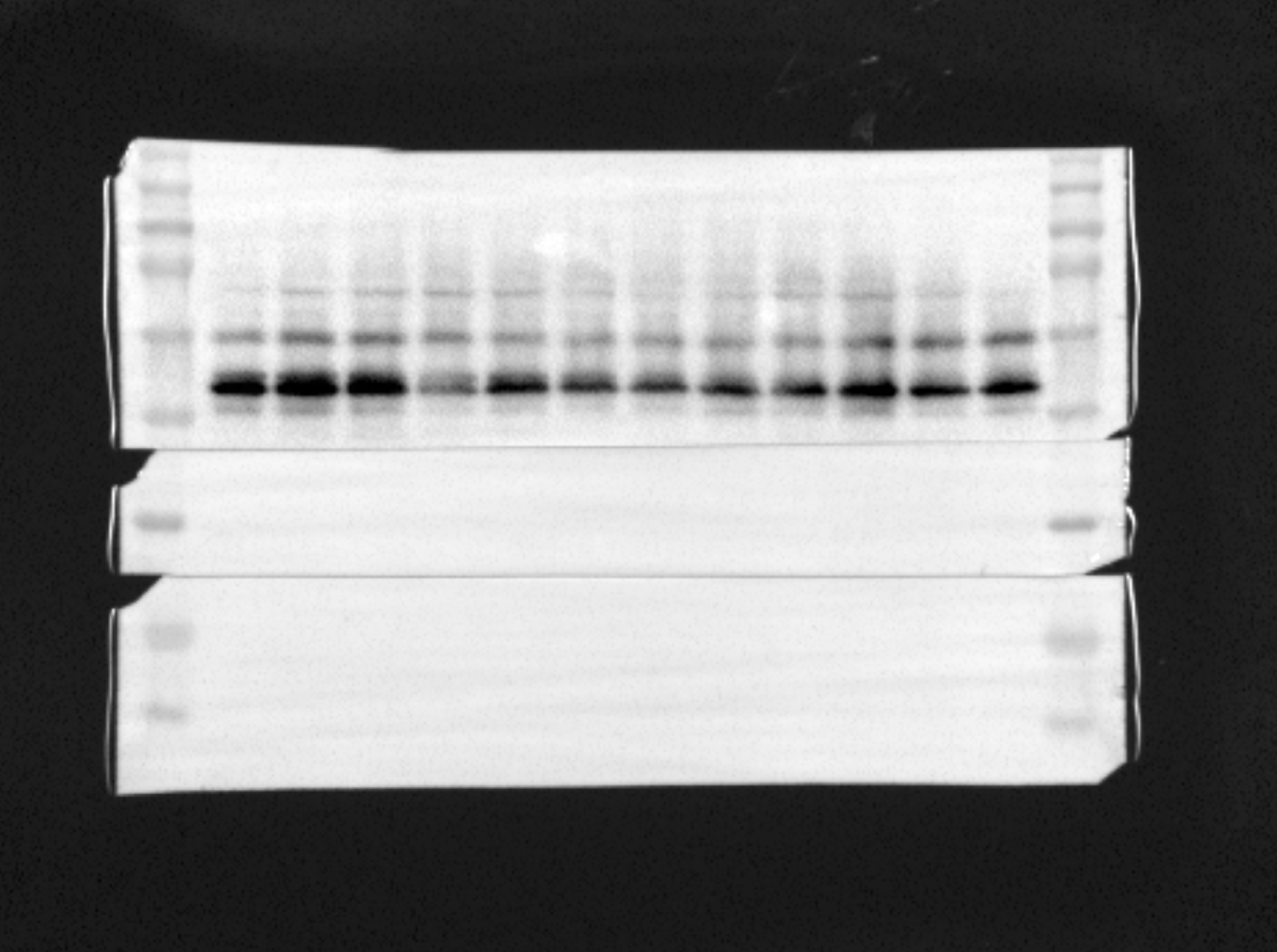

Supplement: Supplementary file 1 — Supplementary Material 1. [file 12958_2024_1250_MOESM1_ESM.zip › WB original picture/fig7-PINK1-parkin-p62-LC3-Actin/parkin/parkin.tif]

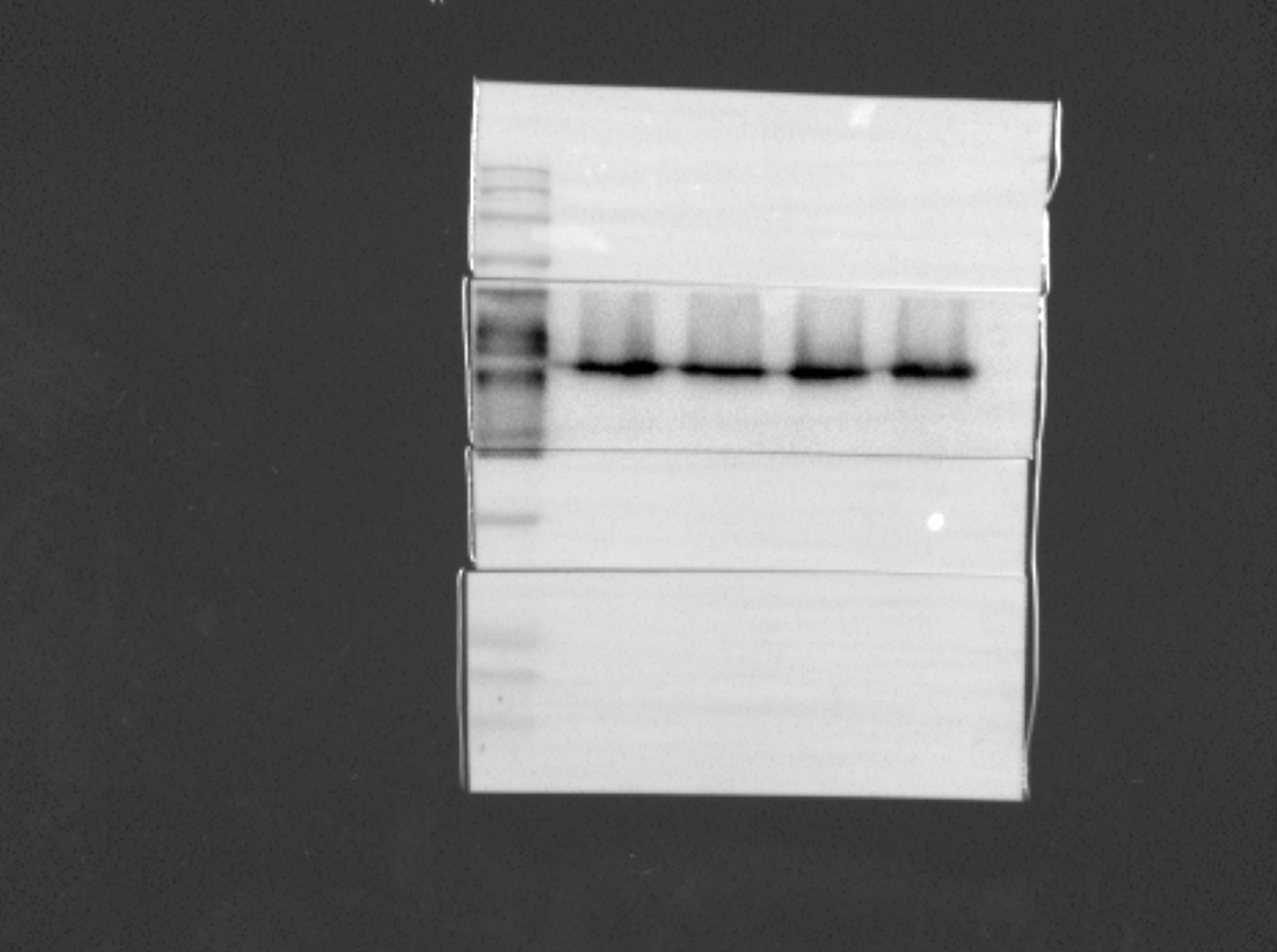

Supplement: Supplementary file 1 — Supplementary Material 1. [file 12958_2024_1250_MOESM1_ESM.zip › WB original picture/fig8-AKT-mTOR/AKT.tif]

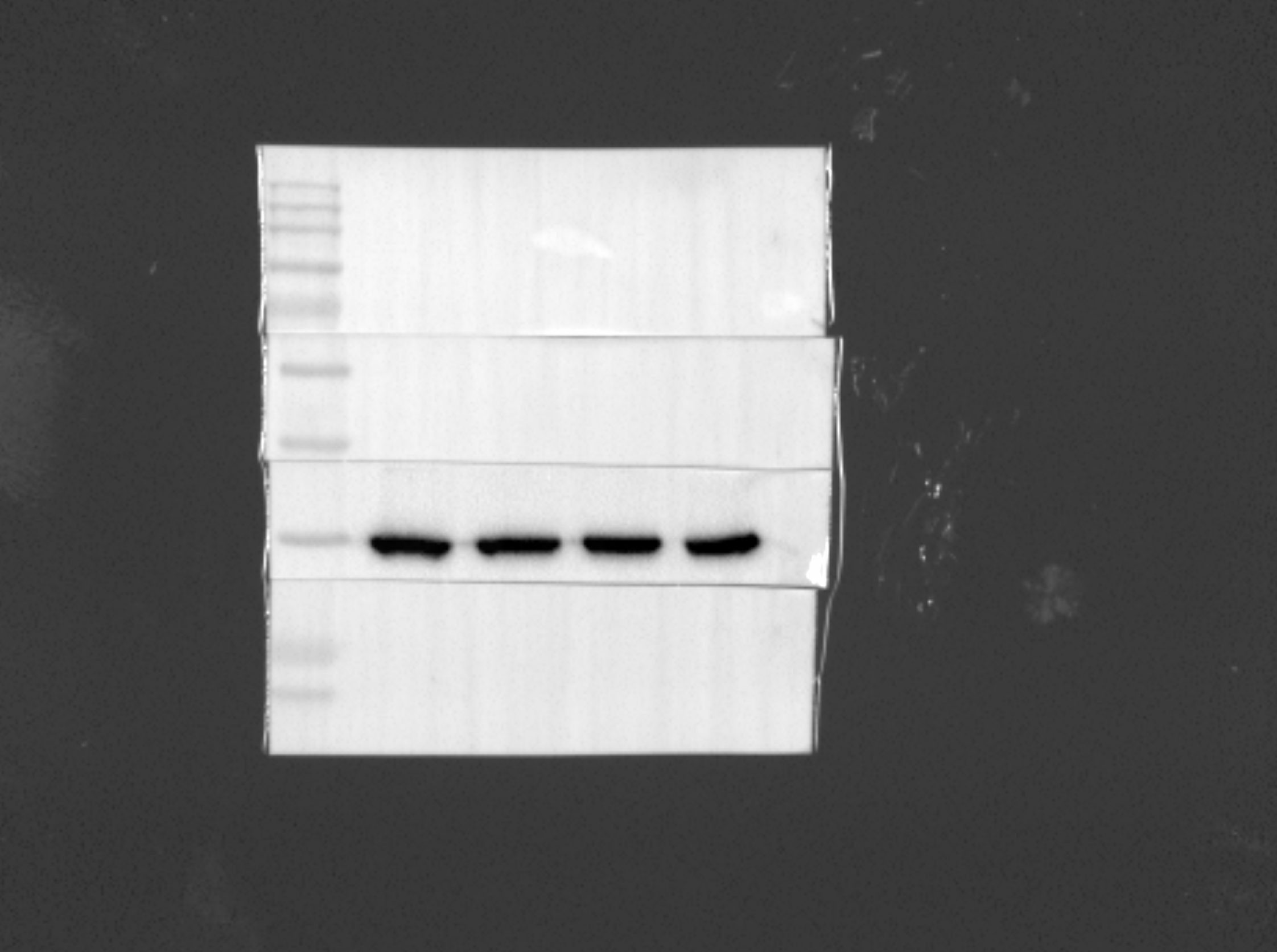

Supplement: Supplementary file 1 — Supplementary Material 1. [file 12958_2024_1250_MOESM1_ESM.zip › WB original picture/fig8-AKT-mTOR/GAPDH.tif]

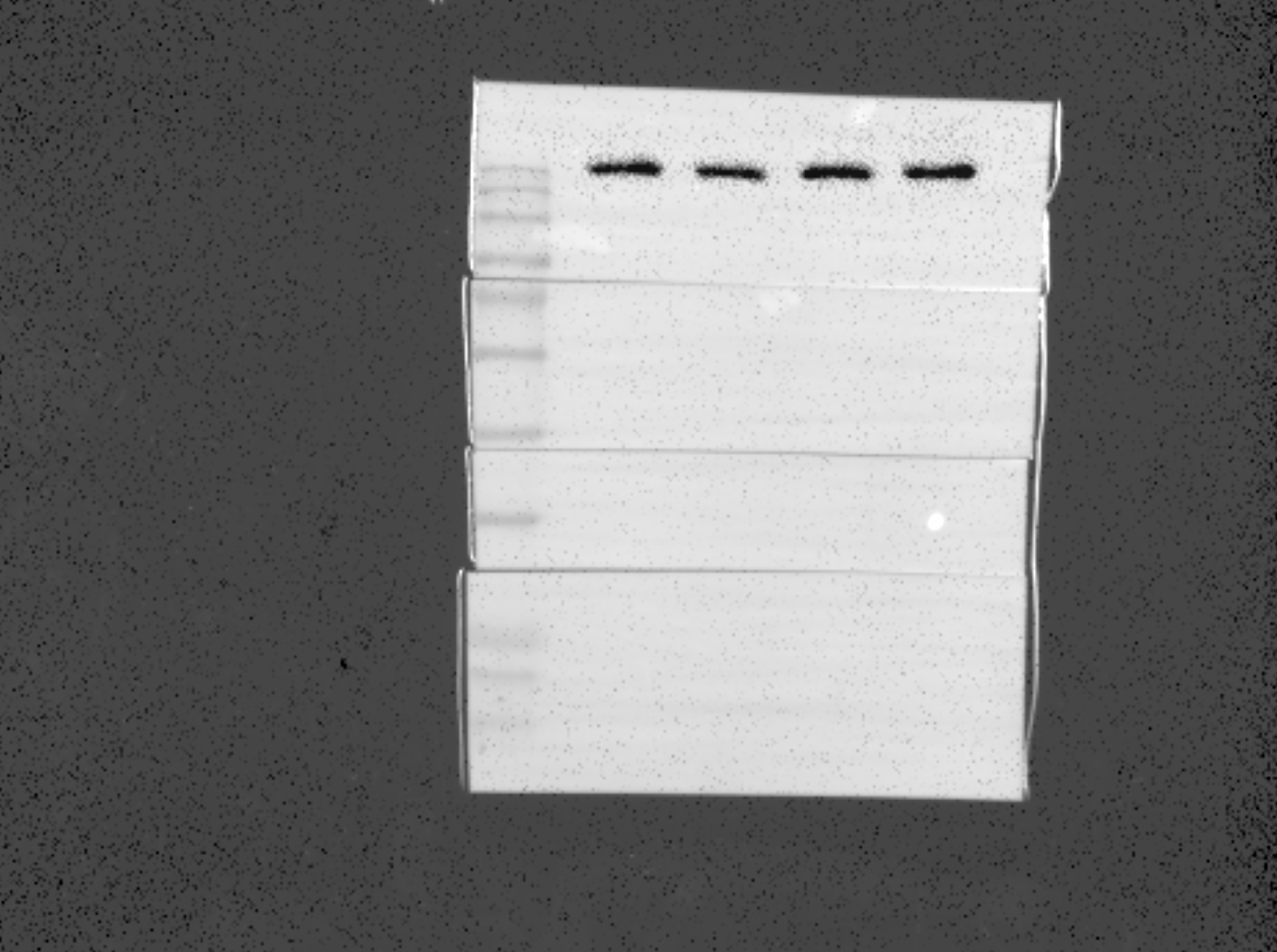

Supplement: Supplementary file 1 — Supplementary Material 1. [file 12958_2024_1250_MOESM1_ESM.zip › WB original picture/fig8-AKT-mTOR/mTOR.tif]

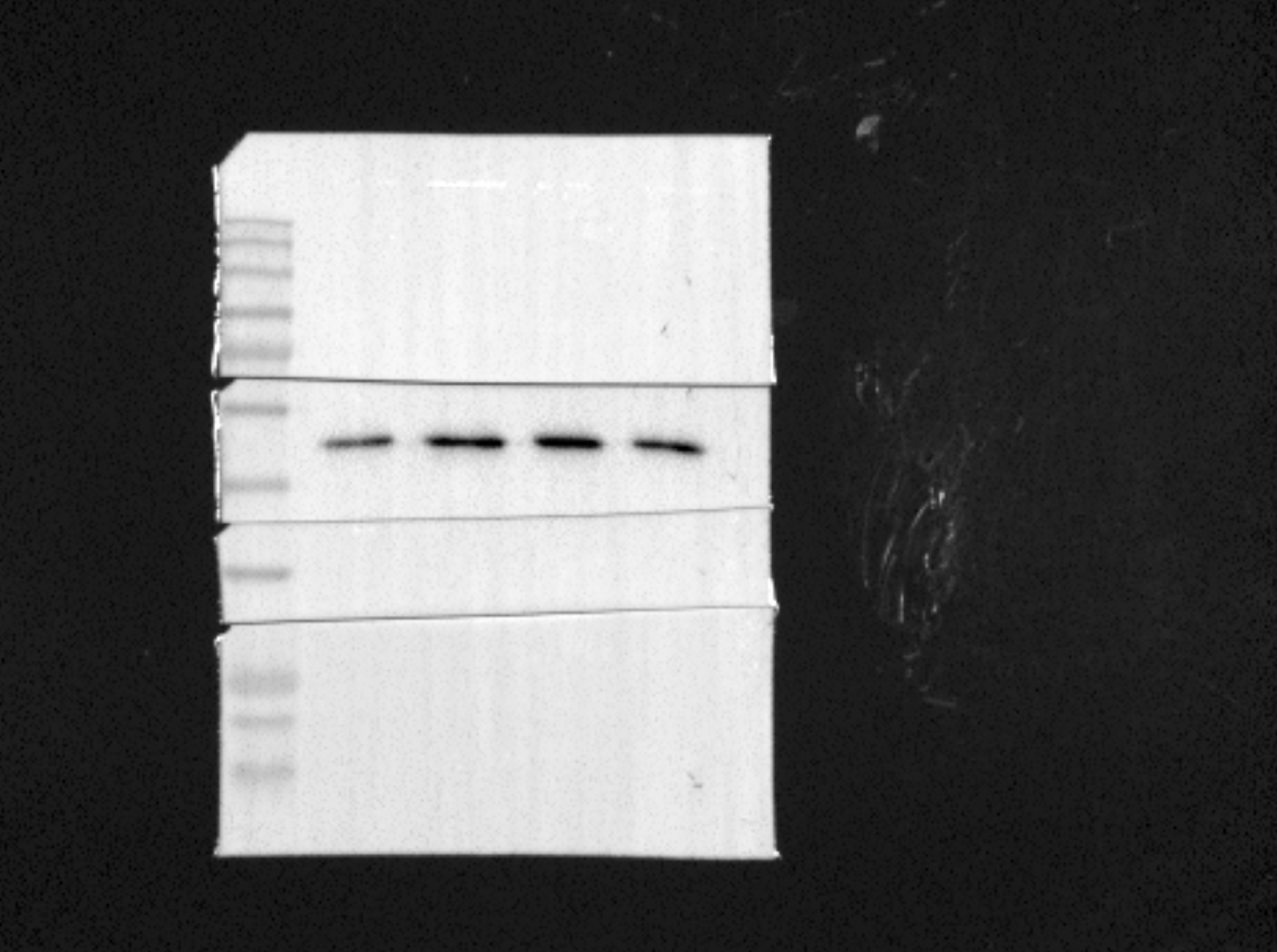

Supplement: Supplementary file 1 — Supplementary Material 1. [file 12958_2024_1250_MOESM1_ESM.zip › WB original picture/fig8-AKT-mTOR/p-AKT .tif]

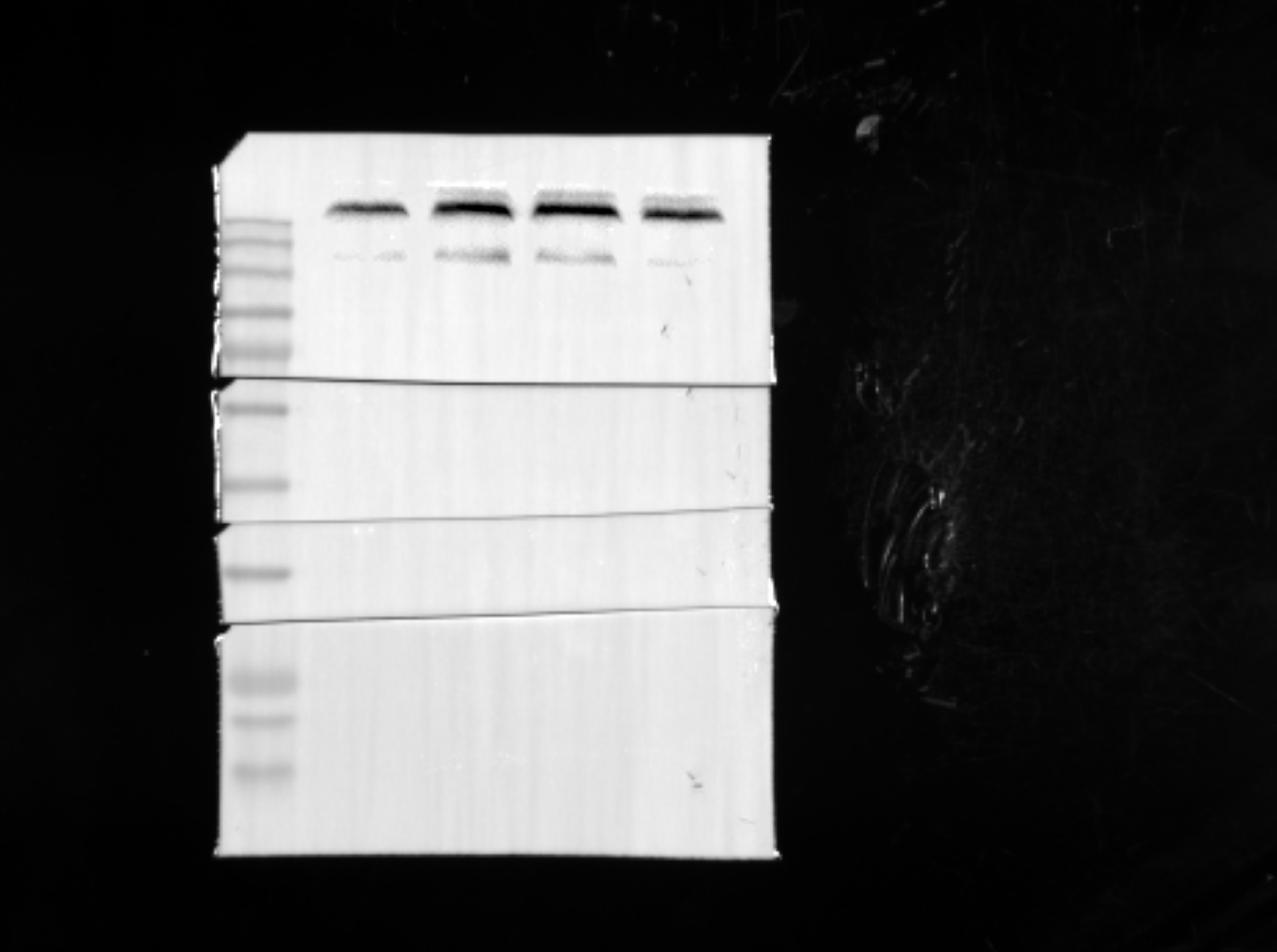

Supplement: Supplementary file 1 — Supplementary Material 1. [file 12958_2024_1250_MOESM1_ESM.zip › WB original picture/fig8-AKT-mTOR/p-mTOR .tif]
